# Exploring Plasmonic Standalone Surface-Enhanced Raman Scattering Nanoprobes for Multifaceted Applications in Biomedical, Food, and Environmental Fields

**DOI:** 10.3390/nano14221839

**Published:** 2024-11-17

**Authors:** Valentina Rojas Martínez, Eunseo Lee, Jeong-Wook Oh

**Affiliations:** Department of Chemistry, Hankuk University of Foreign Studies (HUFS), Yongin 17035, Republic of Korea; valecticarm@gmail.com (V.R.M.); dldmstj0123@hufs.ac.kr (E.L.)

**Keywords:** surface-enhanced Raman scattering (SERS), standalone SERS nanoprobes, SERS nanotags, nanogap enhancement, multiplexed biosensing

## Abstract

Surface-enhanced Raman scattering (SERS) is an innovative spectroscopic technique that amplifies the Raman signals of molecules adsorbed on rough metal surfaces, making it pivotal for single-molecule detection in complex biological and environmental matrices. This review aims to elucidate the design strategies and recent advancements in the application of standalone SERS nanoprobes, with a special focus on quantifiable SERS tags. We conducted a comprehensive analysis of the recent literature, focusing on the development of SERS nanoprobes that employ novel nanostructuring techniques to enhance signal reliability and quantification. Standalone SERS nanoprobes exhibit significant enhancements in sensitivity and specificity due to optimized hot spot generation and improved reporter molecule interactions. Recent innovations include the development of nanogap and core–satellite structures that enhance electromagnetic fields, which are crucial for SERS applications. Standalone SERS nanoprobes, particularly those utilizing indirect detection mechanisms, represent a significant advancement in the field. They hold potential for wide-ranging applications, from disease diagnostics to environmental monitoring, owing to their enhanced sensitivity and ability to operate under complex sample conditions.

## 1. Introduction

SERS is a pivotal technique in spectroscopy that remarkably enhances the Raman signals of molecules adsorbed on rough metal surfaces or nanostructures, typically gold or silver. Since its empirical discovery in the 1970s, SERS has been the subject of intense research due to its ability to detect single molecules through the enhancement of electromagnetic (EM) fields generated at the nanostructure’s surface [1,2,3]. This foundational work paved the way for the subsequent development and refinement of SERS as a robust analytical tool.

**Figure 1:**
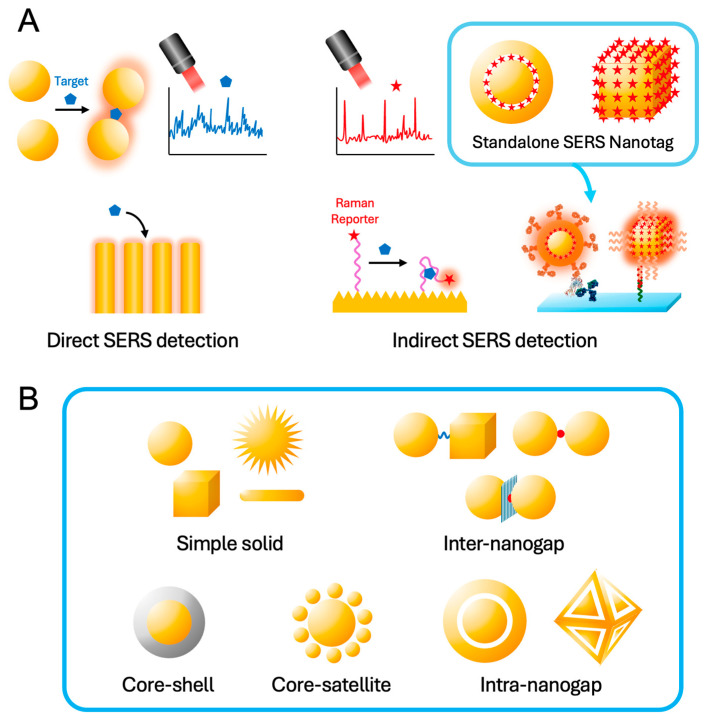
(**A**) SERS-based sensors: Direct SERS detection using the Raman signal of target molecules and indirect SERS detection using the Raman signal transformation of Raman reporter molecules (e.g., Raman reporter modified aptamer) or the amplified Raman signal of standalone SERS nanotags. (**B**) Various nanostructures for efficient standalone SERS nanoprobes.

SERS-based sensors, particularly useful in biological, clinical, and environmental analysis, are generally classified into two main categories: direct and indirect SERS-based sensors [4,5,6,7,8]. As shown in Figure 1, direct SERS-based sensors capture target analytes directly on the SERS active surface, such as elaborately designed plasmonic hot spots, thus obtaining and analyzing the fingerprint information of the target analyte. This straightforward technique, however, faces challenges in biological, clinical, and environmental systems, as follows. The primary limitations include the inherently subtle and complex Raman spectroscopic signatures of biomolecules, a lack of established SERS spectra for various biomolecules and environmental toxins, and the intrinsic complexity of real sample matrices. Furthermore, while reducing the dimensions of hot spots can enhance SERS signal intensity, it simultaneously restricts access to high-molecular-weight biomolecules. This leads to a dilemma where increasing signal strength inversely affects the sensor’s ability to capture target molecules, thus reducing the overall sensitivity of the system.

In contrast, indirect SERS-based sensors utilize the Raman signals of reporter molecules with a high Raman cross-section, which consists of an enhancing substrate, receptors, Raman reporters, and targeting molecules. This configuration not only simplifies the analysis by focusing on the Raman signals of the reporter molecules but also enhances specificity and multiplexing capabilities, making it highly suitable for dynamic biological environments where direct measurements may be difficult.

Additionally, indirect methods often employ SERS nanotags, which are standalone nanoprobes embedding many reporter molecules and modifying receptors on the surfaces for target molecular recognition [9,10,11]. This approach not only enhances the sensitivity and specificity of the detection but also allows for the multiplexed analysis of various substances simultaneously. Standalone SERS nanotags represent a significant advancement within the category of indirect SERS-based sensors via immunoassay platforms. These nanotags are engineered to optimize the Raman signals of reporters and the interaction between the Raman reporter and the nanostructured metal surface (i.e., hot spot), ensuring high sensitivity and specificity. They can be tailored for various applications, from disease diagnostics to environmental monitoring, making them versatile tools in both fundamental and applied sciences. Recent advancements in SERS nanotag research have been driven by the need for rapid and accurate diagnostics during the COVID-19 pandemic and the emerging concerns over environmental issues like microplastics and nanoplastics, highlighting their potential for both medical and environmental applications [12,13,14,15,16,17]. The use of standalone SERS nanotags is particularly beneficial in complex samples where direct SERS applications might suffer from spectral interference or insufficient sensitivity.

Over the years, numerous review papers have thoroughly documented the development and applications of SERS, including seminal works by leading researchers in the field [6,7,10,18,19,20,21]. In this review, we focus specifically on the latest advancements in standalone SERS nanotag technology and the innovative synthetic strategies that have been developed. The purpose of this review article is also to explore the distinctions and applications of direct versus indirect SERS-based sensors, with a special emphasis on the advancements and potential of standalone SERS nanotags in indirect detection. By comparing these strategies, we aim to provide insights into their operational principles, discuss their respective advantages and limitations, and highlight innovative developments that could shape the future of SERS technology. This review aspires not only to serve as a resource for current knowledge but also to inspire further research and development in enhancing the capabilities of SERS-based sensors. Through a detailed exploration of these technologies, we hope to underscore the transformative potential of SERS in tackling analytical challenges across diverse scientific disciplines.

## 2. Basics of SERS Nanoprobes

### 2.1. SERS Effect and SERS Mechanisms

The Raman effect, discovered by Raman in 1928 and independently observed by Landsberg and Mandelstam [22], marked a significant milestone in studying molecular vibrations and chemical compositions by spectroscopy. Despite the limitation of a weak signal, Raman spectroscopy advanced significantly in the 1960s with the advent of lasers as a powerful light source. However, due to the inherently small cross-section of Raman scattering (dσ_R_/dΩ∼10^−31^ cm^2^ sr^−1^) compared with that of fluorescence emission (dσ_F_/dΩ∼10^−16^ cm^2^ sr^−1^), Raman spectroscopy posed challenges in sensitivity and detection limits for practical applications [23].

A pivotal development in the field occurred in the mid-1970s when Fleischmann et al. discovered the SERS effect [1]. They observed a significant increase in the Raman signal from pyridine molecules on roughened silver electrodes. This discovery was further refined by Jeanmaire and Van Duyne [3], as well as Albrecht and Creighton [2], who contributed to understanding the mechanisms behind SERS. By dramatically amplifying the Raman signal using metallic nanostructures, SERS addressed sensitivity issues and enabled single-molecule detection [24,25,26]. This advancement, along with ongoing nanotechnology improvements, has made SERS a powerful analytical tool across various scientific fields.

**Figure 2:**
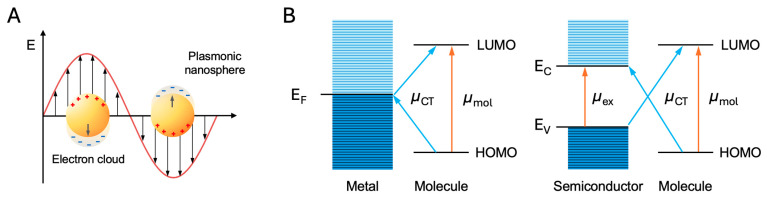
SERS mechanisms: (**A**) LSPR-induced EM enhancement. (**B**) CT resonance mechanism of CM enhancement mechanisms at a metal-molecule or semiconductor-molecule interface. The arrows indicate CT transitions (*μ*_CT_), electronic transitions of a molecule (*μ*_mol_), E_F_ (Fermi level), HOMO (highest occupied molecular orbital), LUMO (lowest unoccupied molecular orbital), VB (valence band), and CB (conduction band).

The enhancement observed in SERS is predominantly attributed to two key mechanisms: the EM mechanism and the chemical (CM) mechanism (Figure 2). The EM mechanism is fundamentally based on the enhancement of the local EM field near the metal surface, induced by localized surface plasmon resonance (LSPR). When the incident light excites the conduction electrons in the metal, these electrons oscillate collectively, leading to a substantial amplification of the local EM field. This field enhancement can lead to Raman signal intensities increased by factors ranging from 10^3^ to 10^6^ [27], depending on the specific conditions of the metal nanostructures and the excitation wavelength. Noble metals such as Au, Ag, and Cu are particularly effective due to their strong plasmonic properties in the visible and NIR regions. The strength of the EM enhancement is highly dependent on the resonance conditions and the morphology of the metal nanostructures, with the enhancement effect rapidly decaying within a few nanometers from the metal surface.

The SERS enhancement factor (EF) *G_SERS_* can be expressed as follows:

(1) $$G_{SERS}\propto {\left|\frac{E_{loc}(\lambda_{ex})}{E_{0}(\lambda_{ex})}\cdot \frac{E_{loc}(\lambda_{em})}{E_{0}(\lambda_{em})}\right|}^{2}$$

Here, *E_loc_* is the local electric field at the nanoparticle surface, *E*_0_ is the incident electric field, *λ_ex_* is the excitation wavelength, and *λ_em_* is the emission wavelength (i.e., Raman-shifted wavelength) [6]. When the excitation and emission wavelengths are close to each other, the enhancement can be approximated by

(2) $$G_{SERS}\propto {\left|\frac{E_{loc}}{E_{0}}\right|}^{4}.$$

The primary contributor to the significantly enhanced SERS signal is the fourth power of the field enhancement in the localized area, which underpins the EM mechanism. For a rigorous calculation, especially when there is a significant difference between the wavelengths, the EM field at each wavelength must be determined separately to compute the SERS EFs accurately. When multiple wavelength lasers are used on the same SERS substrate condition, the approximation in Equation (2) is typically good in the blue and green regions but not for the lower energy wavelength, the red or near-infrared (NIR) regions [20].

To determine the distribution of the EM field near nanostructures, theoretical calculation methods are typically employed. The interaction of light with plasmonic nanoparticles can be described by Mie theory, which provides a comprehensive framework for understanding the scattering, absorption, and extinction of light by spherical particles [28,29,30]. However, Mie theory is only exact for spherical particles and has limitations when applied to more complex nanostructures, such as rods, cubes, and star-shapes. To address these limitations, numerical methods like the discrete dipole approximation (DDA) and finite-difference time-domain (FDTD), and finite-element method (FEM) are used. These methods enable calculating the optical properties of arbitrarily shaped particles by discretizing the particles into smaller elements and solving Maxwell’s equations for each element [31,32]. For more detailed theoretical methods and calculations, readers are encouraged to refer to the specific literature on these techniques [33,34].

The theoretical framework underpinning plasmonic nanoparticles and their application in SERS involves a combination of classical electromagnetic theory and quantum mechanical principles. Classical approaches, such as Mie theory, DDA, and FDTD, provide insights into the interaction of light with nanostructures, while quantum mechanical models account for the discrete electronic states and non-linear optical effects that arise at the nanoscale. For instance, the quantization of plasmonic modes in small nanoparticles leads to phenomena such as plasmon-induced hot electron generation, which can be harnessed for photochemical reactions and sensing applications. Additionally, the interplay between plasmonic nanoparticles and their environment can lead to hybridized plasmon modes, enhancing the local density of optical states and further boosting SERS signals. For the accurate theoretical calculation of the EM field at the sub-nm gap level, the quantum tunneling effect should also be considered [35,36,37,38].

While the EM mechanism is related to the near-field around the plasmonic nanoparticles, the CM mechanism involves direct interactions between adsorbed molecules and the nanoparticle surface. The CM enhancement involves three main types: (1) charge transfer (CT) resonance, (2) adsorption-induced polarizability change, and (3) resonance Raman enhancement. CT resonance involves electron transfer between the metal substrate and the molecule, altering electronic states and enhancing Raman scattering. Adsorption-induced polarizability change happens when a molecule’s polarizability changes upon adsorption to the metal surface, enhancing the signal, especially for nonresonant molecules. Resonance Raman enhancement occurs when the incident light’s wavelength matches a molecular electronic transition, significantly increasing Raman scattering. These mechanisms collectively enhance SERS sensitivity and specificity. The EFs for the CM mechanism are typically smaller, ranging from 10^1^ to 10^3^, but are crucial for molecules that can form specific chemical bonds with the metal surface or molecules adsorbed on a semiconductor substrate with low EM enhancement [27,39,40,41]. The CM enhancement is highly molecule-specific, relying on the electronic structure of the adsorbed molecule and the nature of its interaction with the metal or the energy level of the semiconductor. Factors such as the type of functional groups present on the molecule, the orientation of the molecule on the surface, and the presence of surface defects or contaminants can significantly influence CM enhancement [7,42].

While the EM mechanism provides a broad and substantial enhancement applicable to a wide range of molecules, the CM mechanism offers additional, albeit smaller, enhancements that are highly dependent on the specific interactions between the analyte and the substrate surface. In general, to increase the SERS EF, plasmonic materials such as noble metals aim to maximize the EM effect by elaborately designing nanostructures and hot spots. In contrast, the inherently low carrier density of semiconductors diminishes the intensity of LSPR and shifts the resonance wavelength away from the visible range, thereby reducing the EM effect. Thus, metal oxide materials, which act as semiconductors, focus on matching the energy levels between the adsorbed molecules and the substrate to improve the CM effect.

These two mechanisms often coexist, contributing synergistically to the overall SERS effect. The interplay between EM and CM mechanisms can be exploited to optimize SERS substrates for specific analytical applications, making SERS a versatile and powerful tool for molecular detection and analysis [7,43].

### 2.2. Plasmonic Nanoparticles for SERS Nanoprobes

Plasmonic nanoparticles exhibit unique optical properties due to the excitation of LSPRs. These resonances occur when the conduction electrons on the nanoparticle surface oscillate coherently in response to an incident EM field. The relationship between the LSPR wavelength and SERS intensity is critical in the design of plasmonic nanoparticles. The LSPR wavelength is determined by the nanoparticle’s material, shape, size, and surrounding medium. For maximum SERS efficiency, the LSPR wavelength should coincide with the laser excitation wavelength used in the Raman spectroscopy experiment, maximizing the EM enhancement effect and leading to stronger SERS signals, as described in the previous sub-section [44]. Consequently, the synthetic design of well-tuned plasmonic structures is a crucial initial step toward developing highly efficient SERS nanoprobes.

Designing plasmonic nanoparticles for SERS applications involves optimizing their physical and chemical properties to maximize Raman signal enhancement. Key strategies include material selection, shape control, size optimization, and surface functionalization. Silver (Ag) and gold (Au) are the most commonly used materials for plasmonic nanoparticles due to their superior plasmonic properties in the visible and NIR regions. Other materials, such as copper (Cu) and aluminum (Al), are also explored for specific applications [45]. The shape of the nanoparticles significantly influences their plasmonic properties. Spherical nanoparticles exhibit a single plasmonic peak, whereas anisotropic shapes such as rods, cubes, and stars display multiple plasmonic modes, often providing greater field enhancement and tunability across a broader range of wavelengths. Typically, nanoparticles in the range of 10–100 nm are used for SERS, with the optimal size depending on the specific application and desired wavelength of operation. The surface chemistry of the nanoparticles can be tailored to enhance their stability, biocompatibility, and specificity for target molecules, with common functionalization strategies including coating with thiol-based molecules, polymers, or silica shells to improve dispersion stability and prevent aggregation.

Recently, non-noble metal materials such as magnesium (Mg), Al, titanium nitride (TiN), and gallium (Ga) are emerging as viable alternatives for SERS applications with benefits over traditional noble metals, including lower cost, greater abundance, UV plasmonic behavior, and environmental applicability [46,47,48,49]. However, the SERS efficiency and the structural uniformity required for standalone SERS nanotags are still lacking compared to noble metal-based nanoprobes.

Recent research has focused on developing new materials and nanostructures to further enhance the performance of SERS nanoprobes. Advances include hybrid nanostructures, core–shell nanoparticles, nanogap engineering, and bimetallic nanoparticles [50,51,52,53,54,55]. Hybrid nanostructures combine plasmonic nanoparticles with other materials, such as semiconductors or magnetic nanoparticles, to create multifunctional hybrid nanostructures offering enhanced SERS activity and additional functionalities such as magnetic separation or photocatalysis. Core–shell nanoparticles, with a plasmonic core and a shell of another material such as silica or polymer, improve stability, biocompatibility, and surface functionality and can be engineered to create hot spots at the core–shell interface for enhanced SERS sensitivity. Creating nanogaps or junctions between nanoparticles, often referred to as “hot spots”, where the EM field is significantly intensified, is another recent advancement. Various techniques such as DNA assembly, lithography, and self-assembly are employed to precisely control the spacing between nanoparticles to optimize SERS performance, providing signal enhancement up to 10^10^ to 10^14^ [56,57,58,59]. However, in practice, a SERS EF in the range of 10^7^ to 10^8^ is sufficient for detecting single-molecule SERS events [58,60,61]. Bimetallic nanoparticles, which combine the properties of two different metals, achieve synergistic effects that enhance plasmonic properties and SERS performance. These nanoparticles offer tunable plasmon resonances and improved chemical stability compared to their monometallic counterparts.

The Raman reporter molecule is another crucial component, selected for its strong Raman signal and ability to chemically bind to the metal surface, thereby leveraging both the EM and CM mechanisms. Commonly used reporters include dyes like Rhodamine 6G and small organic molecules. Strong binding of the reporter molecule to the metal surface, often facilitated by thiol groups, ensures stability and reproducibility of the SERS signal.

To enhance the stability and biocompatibility of SERS nanotags, a protective coating or functional layer is often applied. This layer, which can be made of materials such as silica or polymers, protects the reporter molecule from environmental factors and enhances the biocompatibility of the nanotags. Additionally, the surface of the nanotags can be functionalized with biomolecules such as antibodies or peptides, enabling targeted detection and imaging applications. Functionalization enhances the specificity of the nanotags for particular analytes or cells, making them highly effective for use in bioimaging, biosensing, and diagnostics.

Optimization and characterization are critical steps in the development of effective SERS nanotags. Synthetic conditions, such as pH, temperature, and reaction time, must be carefully controlled to achieve uniform and reproducible SERS nanotags. Comprehensive characterization using techniques such as transmission electron microscopy (TEM), scanning electron microscopy (SEM), UV-Vis spectroscopy, and Raman spectroscopy is essential to confirm the size, shape, and surface chemistry of the nanotags. Achieving a practical EF is crucial for the effective application of SERS sensors. Typically, EFs in the range of 10^5^ to 10^8^ are necessary for SERS-based detection and sensing [57,61]. These high EFs enable the detection of analytes at very low concentrations, potentially down to the single-molecule level in some cases. Such levels of enhancement ensure that SERS sensors provide the high sensitivity and specificity required for various analytical and diagnostic applications. By optimizing the interplay between the EM and CM mechanisms, SERS substrates can be designed to achieve these practical EFs, thereby improving the overall performance and applicability of SERS sensors in diverse fields.

## 3. Design for Efficient Standalone SERS Nanoprobes

### 3.1. Shape Controlled Nanoparticles

**Figure 3:**
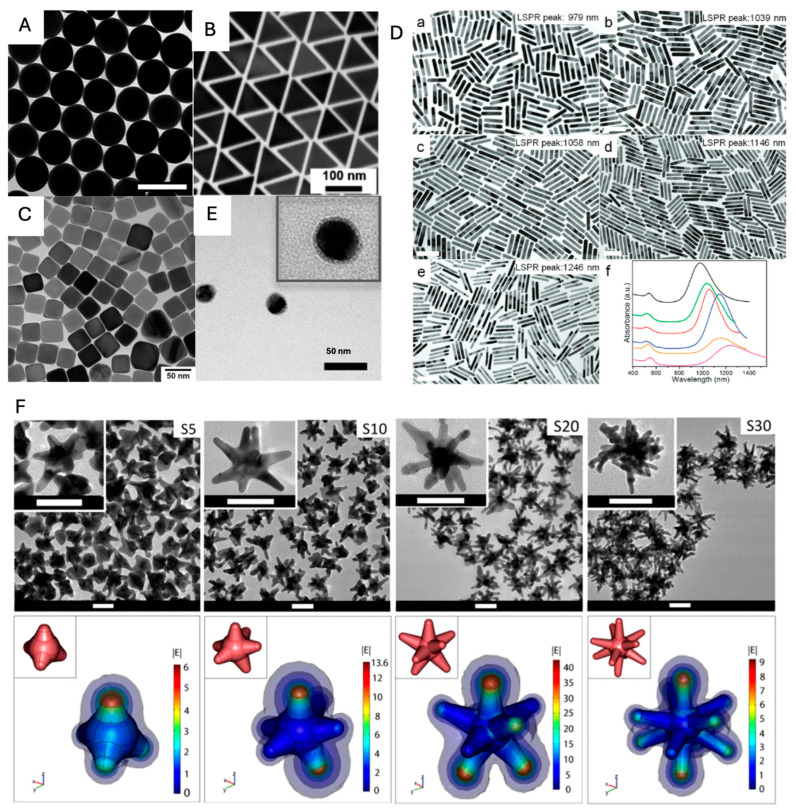
(**A**–**D**) Transmission electron microscopy (TEM) images of diverse morphologies of nanoparticles: (**A**) Au nanosphere (adapted with permission from [68]; Copyright 2021 American Chemical Society). (**B**) Au nanotriangles (adapted with permission from [69]; Copyright 2022 American Chemical Society). (**C**) Au nanocubes (adapted with permission from [70]; Copyright 2022 Elsevier). (**D**) Au nanorods with various aspect ratios. The ratios of Au NRs are 5.9, 6.4, 6.4, 7.5, and 8.5, corresponing to Figures (**D**-**a**) through (**D**-**e**), respectively. (**D**-**f**) UV-vis-NIR spectra of Au NRs shown in (**D**-**a**) (black), (**D**-**b**) (green), (**D**-**c**) (red), (**D**-**d**) (blue), and (**D**-**e**) (magenta), respectively. The orange curve is the UV-vis-NIR spectrum of Au NRs synthesized with the ratio of 7.3. All scale bars represent 100 nm. (adapted with permission from [63]; Copyright 2012 American Chemical Society). (**E**) TEM image of Ag@NO_2_ (adapted with permission from [66]; Copyright 2022 The Royal Society of Chemistry). (**F**) Au nanostars (adapted with permission from [67]; Copyright 2012 IOP Publishing).

As mentioned before, plasmonic nanoparticles generate the SERS signal of adsorbed molecules via the EM mechanism, which varies depending on the shape, size, and composition of each nanoparticle. The “lightning rod effect”, which refers to the phenomenon of concentrating the EM field at the tips or edges of metallic nanostructures in plasmonics, plays a crucial role in maximizing the EM field enhancements necessary for boosting Raman signal intensities. This effect is particularly influential in nanoparticle shapes such as nanotriangles and nanocubes compared to smoother nanospheres, as shown in Figure 3A–C. From the DDA numerical method, the maximized values (confined EM filed at the vertex) of ${\left|E\right|}^{2}$ of three different shapes of nanoparticles (i.e., a sphere, a cube, and a pyramid) were observed on the order ~10^2^, ~10^3^, and ~10^4^, respectively, corresponding to each LSPR peak [62]. In designing standalone SERS nanoprobes, precision in the placement of Raman reporter molecules is paramount, particularly given the greater heterogeneity in EM field distributions. It is crucial to ensure that these reporter molecules are strategically positioned within the zones of amplified EM fields to optimize the SERS effect.

The EM field (i.e., near-field) distributions on nanoparticles are varied by incident light wavelength. For designing efficient SERS nanoprobes, the alignment between excitation light and LSPR band should also be taken into account for maximal SERS enhancement. Anisotropic nanorods have the advantage of fine-tuning the spectral position of LSPR by controlling their aspect ratios (Figure 3D) [63]. For the fabrication of standalone SERS nanoprobes, Raman dyes are typically immobilized onto the surfaces of plasmonic nanoparticles via thiol groups. Additionally, to prevent the detachment of these dyes, they are often encapsulated within a protective coating composed of silica or polymer layers [64,65]. In Figure 3E, a polymerized aryl layer (Ag@NO_2_) is used as a protecting layer, which generates stable SERS signals [66].

Nanoparticles possessing rough surfaces concentrate intense EM fields around their acute tips, thereby yielding enhanced SERS activity in contrast to the more uniformly shaped spherical nanoparticles. The synthesized star-shaped nanoparticles exhibit better SERS effects due to their structurally sharp points (Figure 3F). In general, the sharp-tip morphologies of Au nanostars (Au NSs) are achieved by controlling the concentration of AgNO_3_. As AgNO_3_ is gradually added, starting from low to high concentrations, the synthesized Au NSs develop extremely sharp tips, leading to a pronounced SERS signal [67]. The role of AgNO_3_ (or Ag^+^ ions) in the synthesis of gold nanostars is to assist the anisotropic growth of gold branches on specific crystallographic facets of multi-twinned citrate seeds. Silver ions do not form branches of silver but rather enhance the anisotropic branching of gold by facilitating growth on certain facets. This helps in the formation of nanostars rather than polydisperse rods and spheres, which would form in the absence of Ag^+^.

### 3.2. Inter-Nanogap Nanoparticles

**Figure 4:**
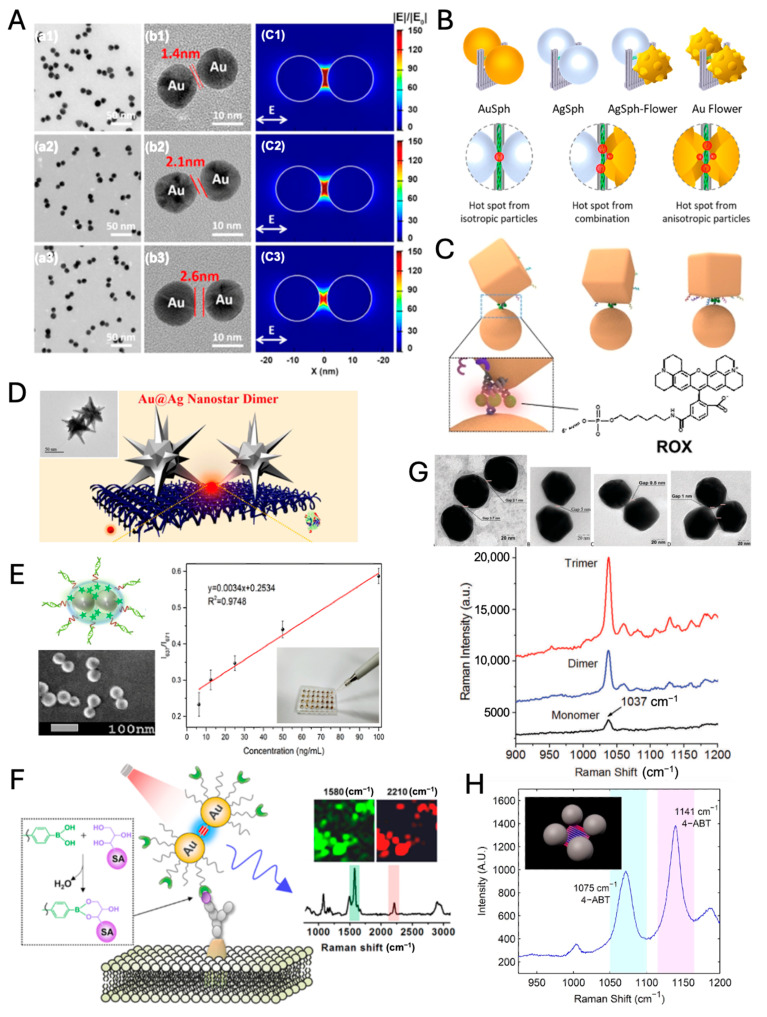
(**A**) Au dimers with nanogaps bridged by metal-organic molecular cages (MOCs) of different sizes (MOC1, MOC2, and MOC3). (**A**-**a**) TEM images, (**A**-**b**) HRTEM images, and (**A**-**c**) simulated electric field distributions around the dimers. **1**, **2**, and **3** corresponds to MOC1, MOC2, and MOC3, respectively. (adapted with permission from REF [76]; Copyright 2021 American Chemical Society). (**B**) DNA origami nanofork-based dimeric structures with various NPs (adapted with permission from REF [79]; Copyright 2023 American Chemical Society). (**C**) Dimeric structure with a nanocube and a nanosphere (adapted with permission from REF [80]; Copyright 2021 Wiley-VCH). (**D**) Au@Ag nanostar dimer (adapted with permission from REF [81]; Copyright 2021 American Chemical Society). (**E**) Detection of endotoxin by SERS chip with dimeric SERS nanotags (adapted with permission from REF [83]; Copyright 2020 American Chemical Society). (**F**) Raman imaging of cancer cells with Au dimers (adapted with permission from REF [84]; Copyright 2017 American Chemical Society). (**G**) Au dimers, trimers, and comparison of their Raman signals (adapted with permission from REF [87]; Copyright 2017 Royal Society of Chemistry) (**H**) DNA origami-based tetramer structure (adapted with permission from REF [86]; Copyright 2014 American Chemical Society).

Nanogaps formed between two or multiple nanoparticles serve as efficient hot spots compared to the surface of an isolated nanoparticle, significantly amplifying the intensity of SERS signals. Dimeric and trimeric nanoparticles with inter-nanogap have been extensively studied for their enhanced EM fields [71,72,73,74,75]. Dimers are relatively simple to produce and integrate into SERS platforms. Additionally, regardless of SERS applications, the precise gap control of plasmonic dimers still attracts much attention due to the gap-dependent characteristic plasmonic modes of dimeric nanoparticles. As the gap distance between two Au nanoparticles (NPs) decreases, the gap plasmon coupling modes become more distinct and red-shifted (Figure 4A) [76]. Additionally, the near-field within the gap becomes increasingly enhanced, allowing highly enhanced SERS signals.

Recently, heterodimeric structures in size, geometry, and composition have been elaborately developed for inducing distinctive optical and physical properties such as dark plasmon modes, Fano resonance (asymmetric line shape), and superior catalytic performance via charge transfer, due to asymmetric plasmonic coupling and symmetry breaking [77,78]. Those dimeric structures, as well as homodimers, can be exquisitely synthesized with precise placement of Raman dyes by DNA origami-based synthetic strategy (Figure 4B,C) [79,80]. Engineering hot spots with nanostar dimers can achieve immense EFs in the range of up to 10^9^–10^10^, enabling the single-molecule detection of thrombin protein (Figure 4D) [81]. Despite the high SERS EFs, the aforementioned dimeric structures are unsuitable for standalone SERS nanotags due to their lack of robustness and stability (e.g., SERS blinking) [82] in biological and clinical conditions.

Ag dimers synthesized with precise control of aggregation were encapsulated with PEG (polyethylene glycol)-thiol to prevent nonspecific adsorption of biomaterials. Those encapsulated Ag dimers were used as SERS nanotags for detecting endotoxin at concentrations as low as 6.125 ng/mL (Figure 4E) [83]. In Figure 4F, alkyne groups were used as Raman dye for profiling of sialic acid expression on the membrane of cancer cells because the alkyne groups have a sharp and distinct peak at 2210 cm^−1^ in cellular Raman silent region (between 1800 and 2800 cm^−1^) [84]. Additionally, a rigid structure of the alkyne group within the nanogap of Au dimers and the post-treatment with PEG-thiols allow robustness in the SERS signal and stability for biological application. In living cells, SERS-based biosensing and bioimaging can also be performed by dynamically assembled nanodimers rather than standalone SERS nanotags. The novel arrowhead Au dimers demonstrated the recognition of miRNAs in living cells, specifically miR-21, through SERS signals [85]. While dynamically assembled nanodimers offer the advantage of detecting target substances within the cytoplasm, the variability of the generated hot spots presents challenges for quantitative analysis compared to standalone SERS nanotags.

Trimers typically provide SERS enhancement stronger than dimers (Figure 4G) due to their multiple hot spots and additional plasmonic coupling [86,87], but they require more sophisticated analysis due to the complexity of their SERS spectra [74,88]. Both dimeric and trimeric nanoparticles can be tailored for specific applications by modifying the particle material, size, shape, and inter-particle distance. To increase the synthesis yield, if necessary, the product is purified by density gradient centrifugation to remove unwanted multimers [89]. In the early days, fundamental investigations into plasmonic dimers were primarily driven by an interest in the correlation between their structure and optical properties. However, recent advancements in synthetic technologies have facilitated their practical application in areas such as biosensing and Raman imaging [83,84,85].

Tetrameric nanoparticle clusters were also developed with precise control of Au NPs using DNA origami structures (Figure 4H) [86,90]. Hot spot position and EM field distribution in multimeric NP structures are affected by the arrangement of NPs and interparticle distance; thereby, the rationally designed multimeric structures and the robustness of constructed multimeric NP assemblies are essential for applying as standalone SERS nanoprobes.

### 3.3. Core–Shell Nanostructures

Core–shell nanostructures play a critical role in enhancing the stability and sensitivity of SERS nanoprobes, enabling their use in applications ranging from biomedical diagnostics to environmental monitoring. These designs typically involve a plasmonic core, such as gold, encapsulated by a functional shell, which modifies the SERS properties and protects the core.

**Figure 5:**
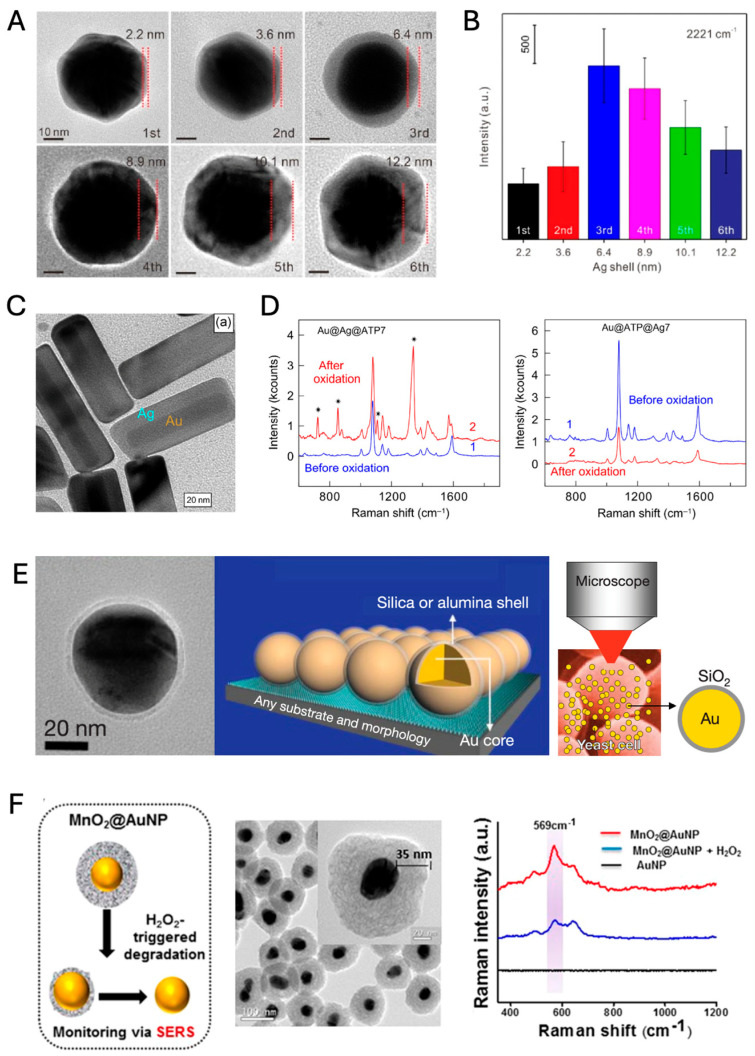
(**A**) TEM images of Au@4-MBN@AgNPs with Ag shell thickness of 2.2, 3.6, 6.4, 8.9, 10.1, and 12.2 nm (adapted under the terms of CC-BY License from REF [91]; Copyright 2024 The Authors, published in Frontiers). (**B**) Raman intensity of different shell thicknesses of Au@4-MBN@AgNPs at 2221cm^−1^ (adapted under the terms of CC-BY License from REF [91]; Copyright 2024 The Authors, published in Frontiers). (**C**) HRTEM images of Au@ATP@Ag nanorods obtained at a sub-threshold 4-ATP concentration CATP = 2.0 × 10^−7^ M (adapted with permission from REF [92]; Copyright 2016 Tsinghua University Press and Springer-Verlag GmbH Germany). (**D**) SERS spectra of the Au@Ag@ATP7 (left) and Au@ATP@Ag7 (right) samples before and after oxidation of the amino groups with hydrogen peroxide. The asterisk represents four additional peaks observed after oxidation, with three peaks at higher wavenumbers corresponding to nitrobenzene (adapted with permission from REF [92]; Copyright 2016 Tsinghua University Press and Springer-Verlag GmbH Germany). (**E**) HRTEM images of Au/SiO_2_ core–shell nanoparticles, SHINERS: shell-isolated mode and schematic of a SHINERS experiment on living yeast cells (adapted with permission from REF [93]; Copyright 2010 Springer nature). (**F**) Schematic representation of H_2_O_2_ triggered degradation of MnO_2_ coating, TEM image, and evaluation MnO_2_ degradation SERS fingerprinting (adapted with permission from REF [94]; Copyright 2021 American Chemical Society).

The Au@Ag core–shell structure is a widely used design that combines a gold core with a silver shell, enhancing the SERS signal intensity [91]. Recent studies have shown that Au@4-MBN@Ag nanoparticles, where 4-mercaptobenzonitrile (4-MBN) is embedded between the Au core and Ag shell, provide significantly stronger SERS signals compared to Au@Ag@4-MBN (4-MBN modified on an Ag shell) and Au@4-MBN (without an Ag shell). The thickness of the Ag shell is a key parameter, as demonstrated in Figure 5A,B, where the optimal thickness for maximum SERS enhancement was identified. This configuration’s enhanced sensitivity and robustness enable it to provide a reliable internal standard Raman signal for the quantitative detection of target molecules.

Au@Ag core–shell nanorods extend the benefits of core–shell designs by incorporating an anisotropic nanorod shape, which enhances plasmonic behavior in the near-infrared (NIR) region. This spectral range aligns with the tissue transparency window, making these nanoprobes suitable for deep-tissue imaging. Monodisperse Au@Ag nanorods with a controlled Ag shell thickness were synthesized, embedding the Raman reporter 1,4-aminothiophenol (4-ATP) between the Au core and Ag shell (Figure 5C) [92]. Figure 5D shows that compared to Raman dye-coated core–shell structures (Au@Ag@ATP), dye-embedded core–shell structures (Au@ATP@Ag) demonstrate resistance to oxidation under hydrogen peroxide treatment, retaining the integrity of the 4-ATP signal. This oxidation-resistant design is crucial for applications in oxidative environments, such as biomedical imaging, where long-term stability is necessary.

Shell-isolated nanoparticle-enhanced Raman spectroscopy (SHINERS) represents another advancement, using a plasmonic core—usually Au—encapsulated by an ultrathin, chemically inert silica shell [93]. This design isolates the core from direct contact with analytes, allowing for non-invasive probing. SHINERS is particularly valuable in in situ biological applications, as illustrated by its use in probing yeast cells. Figure 5E shows how SHINERS enables clear detection of mannoprotein signatures from the cell wall without interference from the surrounding biological environment, underscoring its utility in real-time monitoring of biomolecular dynamics in cells. This approach is also beneficial in fields like food safety and environmental analysis, where complex matrices often obscure traditional SERS measurements.

Finally, MnO_2_@AuNP (where MnO_2_ serves as the shell and AuNP as the core, although the notation “shell@core” is less commonly used) is a novel core–shell structure designed specifically for theranostic applications in cancer treatment [94]. The MnO_2_ shell around an Au core enhances biocompatibility and provides a unique response to oxidative environments. The MnO_2_ shell inherently exhibits a Raman signal at 569 cm^−1^, eliminating the need for additional Raman reporter molecules. In pancreatic cancer cells, the MnO_2_ shell degrades in response to elevated hydrogen peroxide levels, characteristic of tumor microenvironments. This degradation exposes the Au core, allowing real-time SERS tracking of therapeutic effects and enabling photothermal therapy (PTT). As shown in Figure 5F, the degradation of the MnO_2_ shell under H_2_O_2_ conditions leads to a decrease in the MnO_2_ Raman signal. The MnO_2_@AuNP structure’s dual functionality as a diagnostic and therapeutic tool demonstrates its potential in targeted cancer treatments.

### 3.4. Core–Satellite Nanostructures

Core–satellite nanostructures have earned significant interest for their ability to enhance and amplify SERS signals [95]. The core–satellite arrangement incorporates multiple satellites around a single core, significantly intensifying the local electric field distribution in the neck region or junction between the central core and surrounding satellites or between satellites themselves [96,97]. The core–satellite architectures, distinguished by their isotropic nature relative to dimeric or trimeric forms, ensure a consistent EM field distribution irrespective of varying polarization angles of incident light. This attribute facilitates the generation of uniform signals, thereby conferring an advantage in the quantitative analysis of SERS measurements.

The inherent versatility of core–satellite nanostructures presents a plethora of opportunities for material customization, potentially providing multifunctionality with different functional materials for both core and satellite components [98]. It is feasible to precisely manipulate the size, shape, inter-particle spacing, and spatial distribution among the constituent elements, such as cores and satellites, or between individual satellites themselves. This capacity for fine-tuning facilitates the modulation of surface plasmon resonance peaks for targeted applications [95,98,99].

**Figure 6:**
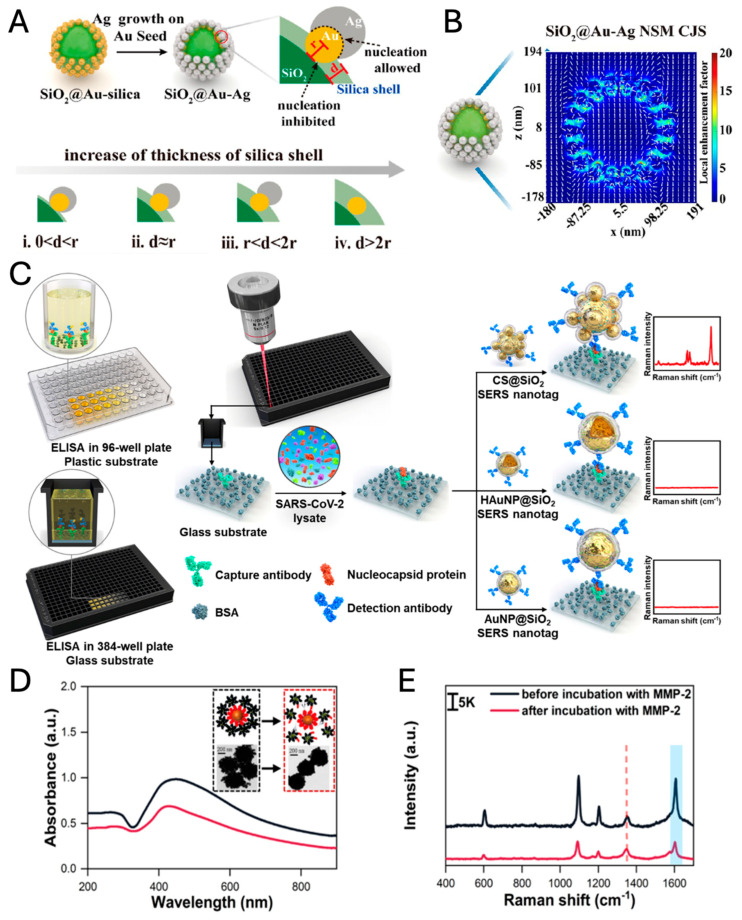
Synthetic schematic diagram (**A**) and electric field distribution (**B**) of SiO_2_@Au-Ag CJS (adapted with permission from REF [97]; Copyright 2023 American Chemical Society). (**C**) Schematic diagram of SERS-ELISA platform with CS@SiO_2_ core–satellite Au NPs (adapted with permission from REF [100]; Copyright 2023 Elsevier). UV-vis spectra, TEM images (inset) (**D**), and SERS spectra (**E**) of the nanosensor before and after incubation with MMP-2. The characteristic peaks of DTNB (5,5′-dithiobis(2-nitrobenzoic acid)) at 1324 cm^−1^ (red dash) and MBN (4-mercaptobenzonitrile) at 1580 cm^−1^ (blue range) (adapted with permission from REF [103]; Copyright 2024 American Chemical Society).

As an illustration of the criticality of manipulating core–satellite ratios, Hao et al. developed a novel approach by introducing a core-Janus satellite (CJS) system, employing Au-Ag Janus structures as satellites with a silica sphere as the core [97]. Here, the silica core does not contribute to the plasmonic coupling between the core and satellites due to its dielectric (i.e., non-plasmonic) nature. However, it provides a solid and inert supporter that maintains the spatial configuration of the Janus satellites. After the formation of the Au-SiO_2_ core–satellite structure, the additional silica shell plays a crucial role in fabricating various Au-Ag Janus structures, which are controlled by the thickness of the silica shell (Figure 6A). An intermediate silica shell (ii. d~r in Figure 6A) enables the formation of nanosnowman (NSM) Janus structures, which generate a strong electric field in their neck region (Figure 6B). The enhanced SERS signal allows for ultrasensitive detection of carbohydrate antigen 19-9, a biomarker of pancreatic cancer, with a detection limit of 3.7 × 10^−5^ IU/mL (IU: international unit).

Core–satellite nanoparticles can be synthesized using various methods, including top-down nanolithography and bottom-up self-assembly. The predominant method for synthesized core–satellite nanostructure is bottom-up self-assembly. This methodology involves connecting cores with the smaller satellites using linkers such as DNAs, proteins, molecules, metal ions, and Raman reporters [100]. These linkers facilitate electrostatic attraction between charged cores and satellites, allowing for covalent bonding, electrostatic adsorption, click chemistry, and other reactions. The advantage of this strategy is that there are no necessary expensive tools, and core–satellite structures can be synthesized through facile chemistry routes [95,101]. Raman reporters as linker molecules between a core and satellites can generate intensive Raman signals due to the significant EM enhancement within nanogaps as hot spots, especially during the core–satellite assemblies [100,102]. Consequently, core–satellites exhibited the greatest SERS signal compared to Au NPs and hollow Au NPs; moreover, the silica shell prevented the aggregation of nanostructures. The sensitivity of the SERS-ELISA (enzyme-linked immunosorbent assay) with CS@SiO_2_ nanotags surpasses that of lateral flow assays (LFAs) and conventional ELISA by factors of 100 and 10, respectively (Figure 6C).

Chen et al. developed a core–satellite structured nano-sensors for the detection of metalloproteinase 2 (MMP-2), the capture core (magnetic bead (MB)@SiO_2_@Au@peptide) and as a satellite (Ag@4-mercaptobenzonitrile (MBN)@ PEG-NH_2_), in which the peptides act as bridges between the capture core and the signal satellites. The capture core structures (Au-coated MBs) play three roles in this system: (1) magnetic probes (MB core) enabling magnetic separation for the determination of Raman signal change, (2) internal standard Raman probes by 5,5′-dithiobis(2-nitrobenzoic acids) (DTNBs) embedded in Au shell, and (3) Raman signal enhancers amplifying the EM-field between Au shell of the capture core and satellites. Interaction between MMP-2 and the substrate peptide induces a conformational collapse of the core–satellite structures, resulting in a measurable reduction in SERS intensity, which provides a sensitive quantification of MMP-2 activity as low as 2.067 ng/mL (Figure 6D,E) [103].

### 3.5. Intra-Nanogap Nanoparticles

Nanogaps formed between two or multiple nanoparticles serve as efficient hot spots compared to the surface of an isolated nanoparticle, significantly amplifying the intensity of SERS signals. Although the fabrication of nanogaps through the aggregation of nanoparticles is particularly advantageous for the dynamic detection of target analytes, it is less ideal for the construction of standalone SERS nanoprobes. Conversely, when a nanogap, serving as a hot spot, is engineered within a single nanoparticle, it lends itself to being an independent SERS nanoprobe. Nanoparticles featuring intra-nanogap structures provide inherent stability and robustness, which ensures the generation of consistent, intense, and quantifiable SERS signals.

**Figure 7:**
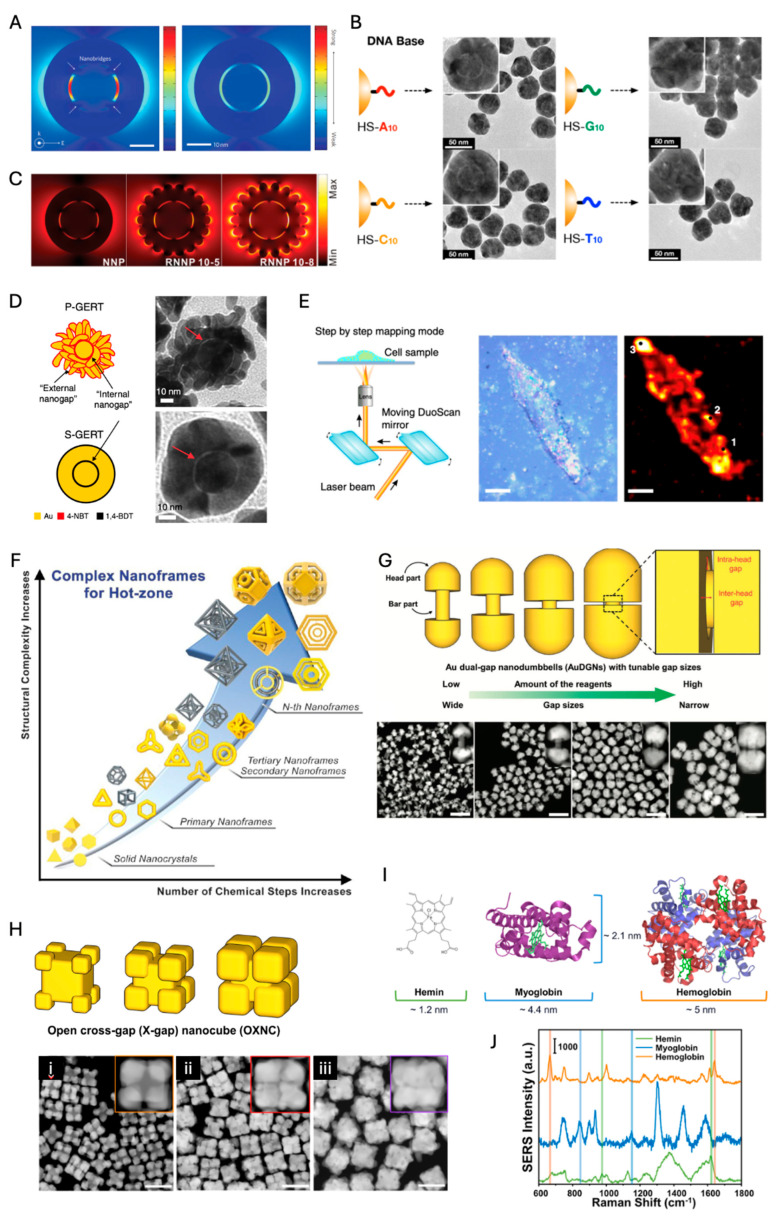
(**A**) Calculated near-field EM field distribution of the Au-NNP and a silica-gapped Au-Au core-gap-shell nanoparticle without a bridge (adapted with permission from [104]; Copyright 2011 Springer Nature). (**B**) TEM images of Au-NNP structures after Au shell formation on various DNA-modified Au cores (adapted with permission from [105]; Copyright 2014 American Chemical Society). (**C**) Calculated near-field EM field distribution of Au-NNPs with different surface morphologies (adapted with permission from [107]; Copyright 2016 Wiley-VCH). (**D**) P-GERTs and S-GERTs (adapted with permission from [110]; Copyright 2019 Springer Nature). (**E**) Schematic diagram of high-speed cell Raman imaging and bright-field and Raman images of a single H1299 cell with different parts randomly selected (point 1–3). Scale bars are 10 μm (adapted with permission from [110]; Copyright 2019 Springer Nature). (**F**) Progression of structural complexity in nanoframes with increasing chemical steps (adapted with permission from [114]; Copyright 2023 American Chemical Society). (**G**) Synthetic scheme and TEM images of AuDGNs (adapted with permission from [115]; Copyright 2016 Wiley-VCH). (**H**) OXNCs with different gap sizes and those HAADF-STEM images (i–iii). The scale bars indicate 100 nm (adapted with permission from [113]; Copyright 2024 American Chemical Society). (**I**) Structures and sizes of hemin, myoglobin, and hemoglobin (adapted with permission from [113]; Copyright 2024 American Chemical Society). (**J**) SERS spectra of hemin (green line) mixed with the OXNC with 2.6 nm gaps, myoglobin (blue line) with the OXNC with 5.6 nm gaps, and hemoglobin (orange line) mixed with the OXNC with 5.6 nm gaps (adapted with permission from [113]; Copyright 2024 American Chemical Society).

Nam et al. invented Au-nanogapped nanoparticle (Au-NNP) structures by Au shell formation on DNA-functionalized Au NPs. DNA bases play a critical role in forming around 1 nm intra-nanogap (Figure 7A). The SERS EF of Au-NNPs becomes eight orders of magnitude, which is enough for single molecule detection [104,105]. The enormous EM enhancement within intra-nanogap results in such strong SERS enhancement. Poly-adenine and poly-cytosine bases on Au core NPs enable the formation of intra-nanogap more clearly than poly-guanine and poly-thymine, which is attributed to the strong binding affinity of adenine and cytosine to the gold surface (Figure 7B). Because the surface coverage of DNA bases is critical to forming intra-nanogap, DNA grafting density should be controlled by the salt-aging process [105,106]. Since Au-NNP structures are isotropic nanoparticles with robust nanogap, they are promising for biomedical applications as reliable, quantifiable standalone SERS nanoprobes [21,105,107]. Interestingly, when the exterior surface of Au-NNP is designed as a rougher surface, the EM field is enhanced within the intra-nanogap as well as the exterior rough surface, which leads to the increased SERS signal by one order of magnitude (Figure 7C) [107].

Ye et al. designed intra-nanogap nanoparticles with benzene thiol-modifiers instead of thiolated DNAs; those intra-nanogap nanoparticles are named GERTs (gap-enhanced Raman tags) [108,109,110]. The gap spacer molecules between the core and shell are smaller aromatic molecules such as 1,4-benzenedithiol (1,4-BDT) and 4-nitrobenzenethiol (4-NBT) than oligo-DNAs, which results in ~0.7 nm interior gap [110]. In these sub-nm intra-nanogap nanoparticles, quantum plasmonic effects via molecular junctions were observed, consistent with the result of quantum-correlated simulation. In contrast to 1,4-BDT, 4-NBT molecules generate dual gaps (i.e., a hollow internal nanogap and petal-like external nanogaps), and such particles were called P-GERTs (smooth external shell structures by 1,4-BDT, S-GERTs) (Figure 7D). Due to the dual gap effect of P-GERTs, the Raman intensity of P-GERTs is larger than that of S-GERTs by two orders of magnitude with a 638 nm laser, achieving SERS EFs beyond 5 × 10^9^. P-GERT nanotags enabled high-speed and high-contrast SERS imaging, capable of obtaining a Raman image of 50 × 50 pixels within 6 s (Figure 7E). This capability is particularly advantageous for intraoperative guidance during surgeries, especially for cancer detection and sentinel lymph node (SLN) identification. Additionally, NIR three different alkyne-embedded intra-nanogap nanoparticles (orthogonal-GERTs, O-GERTs) provide distinct interference-free Raman signals (2000~2300 cm^−1^) in the Raman-silent region, and those were utilized as anticounterfeiting encoding materials [111].

Nanomatryoshkas with multiple intra-nanogaps provide Raman intensity stronger than the single-shell nanomatryoshkas, but most Raman signals of multi-shell nanomatryoshkas are attributed to the outer nanogap, which means the multi-shell effect is not significant [112]. However, when two different Raman reporter molecules are embedded in each intra-nanogap, those SERS nanoprobes can improve encoding capability for multiplexed bioimaging.

Park et al. have recently developed novel 2D and 3D nanoframes with rationally designed intra-nanogaps called “hot zones” confining intensely localized EM fields (Figure 7F). Even though various structures appear complex, there are straightforward synthetic strategies and evolution steps, as follows: (1) synthesis of Au template structure, (2) rim-preferential Pt deposition, (3) selective etching, (4) Au regrowth, and (5) nth repetition for hierarchical and nested structures. Au nanorings and nanoframes regrown from Pt skeletons achieve high SERS enhancement factors EFs exceeding 10^8^. Furthermore, advanced 2D and 3D nanoframes, such as circular, triangular, hexagonal, and Y-shaped hot zones, achieve SERS EFs in the range of 10^9^ to 10^10^. The development of hierarchical and nested structures, like dual-rim faceted nanoframes and frame-in-frame (matryoshka) geometries, enables the maximization of plasmonic coupling within the nanostructure, leading to significantly enhanced Raman signals, with SERS EFs reaching up to 10^11^.

Another recent advancement in intra-nanogap technology is the development of Au dual-gap nanodumbbells (AuDGNs), which utilize anisotropic adsorption of polyethyleneimine (PEI) on gold nanorods (Au NRs) to achieve tip-selective gold growth. This process results in dual nanogaps—inter-head and intra-head gaps—within a single particle, significantly enhancing the surface-enhanced Raman scattering (SERS) signals (Figure 7G). AuDGNs are synthesized with high yields exceeding 90%, and their SERS EFs range from 1.5 × 10^8^ to 1.5 × 10^9^ for over 90% of the particles.

Recently, open cross-gap (X-gap) nanocubes (OXNCs) have been synthesized to produce highly enhanced, reliable, and uniform SERS signals [113]. These structures represent an intermediate form among inter-nanogap, core–satellite, intra-nanogap architectures (Figure 7H). Notably, OXNCs enable label-free detection of biomolecules due to their open nanogap design. In contrast, ordinary intra-nanogap particles contain the nanogap within the nanostructures, making them less suitable for directly determining the Raman fingerprint of target molecules. However, OXJNCs have successfully detected biomolecules of varying sizes, such as hemin, myoglobin, and hemoglobin, by controlling the thickness of the X-gap (Figure 7I,J). Smaller gaps (2.6 nm) are better suited for detecting hemin, while larger gaps (5.6 nm) are more effective for hemoglobin.

The advantages, disadvantages, and optimization strategies of the various nanostructures mentioned above for standalone SERS nanotags are summarized in Table 1.

## 4. Real Application

### 4.1. Clinical Application

The diagnosis of diseases at an early stage is crucial for improving healthcare outcomes and reducing mortality rates while minimizing treatment costs. However, achieving rapid, specific, and sensitive detection of biomarkers in complex biological media remains a significant challenge [116]. Among the emerging technologies, Raman spectroscopy stands out for its potential in disease diagnosis applications. Its integration of high specificity, minimal sample requirements, and advanced technology makes it a valuable tool for qualitative and quantitative detection of various diseases [117]. SERS has become a particularly promising technique for directly detecting biomarkers in disease diagnosis and for in vivo imaging of multiplexed target molecules. This approach has gained significant attention in bioanalysis, especially for detecting analytes in biofluid samples. Researchers have extensively investigated the use of SERS nanotags for this purpose, which provide specific Raman spectra indicating the presence of DNA, proteins, and other biomarkers [118].

#### 4.1.1. Point-of-Care (POC) Diagnostics

The development of point-of-care (POC) analytical tools based on biosensors has emerged as a critical area of research, driven by the need for faster and more accessible diagnostic methods for specific pathologies. Portable biosensors offer the promise of rapid testing and diagnosis, which is particularly important in various healthcare settings. Diagnostics SERS nanoprobes offer remarkable sensitivity and specificity, enabling the detection of minute biomolecular signatures directly in complex samples like blood, sweat, urine, and even breath. This technology allows for non-invasive analysis without the need for extensive sample preparation or complex equipment.

Among these emerging technologies, SERS nanotags-based paper lateral flow strips (PLFSs) have gained attention for their quick testing time, low cost, low limit of detection, ease of operation, and portability across a versatile range of applications [119]. Traumatic brain injury (TBI) is a significant global health concern, often leading to death or permanent disability, especially in low and middle-income countries. Diagnosis of TBI typically relies on the Glasgow Coma Scale (GCS) and neuroimaging techniques, but the use of POC devices could improve early diagnosis and intervention. Sun et al. devised SERS nanotags-based PLFSs with Au nanorods capable of detecting glial fibrillary acidic protein (GFAP) and myelin basic protein (MBP), common biomarkers associated with TBI pathology. The assay methodology facilitates the detection of analytes at lower concentrations, with a limit of detection (LOD) reaching the order of ~10^−1^ pg·mL^−1^, which is significantly lower than that of conventional ultrasensitive ELISA kits [119].

**Figure 8:**
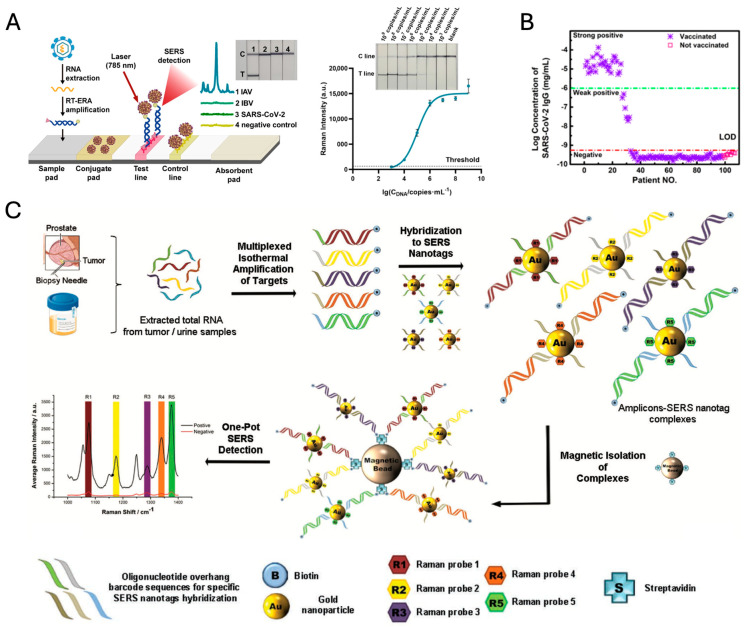
(**A**) ERA-SERS-LF strip (left) and phototgraphs (right, inset) and the calibration curve (Right) of SiO_2_@Au-based ERA-LF-SERS strips when testing the IAV DNA (adapted with permission from [120]; Copyright 2023 American Chemical Society). (**B**) Clinical serum sample tests by Ag@Au NP-based dual-mode LFIA (adapted with permission from [121]; Copyright 2022 American Chemical Society). (**C**) Schematic representation of assay. Total RNA is first isolated from samples before target RNA biomarkers are simultaneously amplified using isothermal reverse transcription-recombinase polymerase amplification. During amplification, amplicons are tagged with biotin molecules and target-specific overhang hybridization sequences. The different biomarker-specific amplicons are then labeled with respective SERS nanotags through complementary sequence hybridization and magnetically purified. Finally, the amplicons are detected by SERS concurrently, and quantitative analysis of biomarker level is derived from the spectral peak of each unique SERS nanotag. The Raman signals correspond to characteristic peaks from the five different dyes of the SERS nanotags, respectively. (Adapted with permission from [126]; Copyright 2016 Wiley-VCH).

A further notable application of SERS nanotags-based PLFSs lies in the identification of infectious pathogens, exemplified by the Influenza A virus (IAV), a microorganism that presents considerable public health challenges owing to its swift rate of transmission. Dong et al. demonstrated an integrated enzymatic recombinase amplification (ERA)-SERS-based lateral flow (LF) strip assay with SiO_2_@Au core–shell nanoraspberries SERS nanotags, designed for the quantitative assessment of IAV [120]. The novel assay manifests a sensitivity threshold detectable by the naked eye at 10^5^ copies/mL and has attained an LOD at 2.63 × 10^3^ copies/mL for IAV gene DNA standards via SERS detection. This represents a significant enhancement in detection capability, approximately 40-fold lower than that observable through visual inspection, attributable to the amplification effects of SERS tagging. Moreover, the assay demonstrates discernment capabilities between Influenza A virus, Influenza B virus, and SARS-CoV-2 pseudoviruses, thereby underscoring the adaptability and prospective influence of PLFS technology within the realm of infectious disease diagnostics (Figure 8A).

Colorimetric and SERS dual-mode lateral flow immunoassay (LFIA) platforms employing 4-mercapto benzoic acid embedded Ag@Au core–shell SERS nanotags were utilized for the detection of severe acute respiratory syndrome–coronavirus-2 immunoglobulin G (SARS-CoV-2 IgG), which serves as a critical index for the diagnosis and monitoring of the progression of SARS-CoV-2 infection. This technique utilized a diagnostic method for SARS-CoV-2 IgG in serum from 107 volunteers, categorizing results as “strong positive”, “weak positive”, or “negative” based on the visibility of the test line, with weak positives confirmed via Raman spectrometry. Among vaccinated subjects, 26 showed strong positive results, 6 weak positive, and 66 negative, while all unvaccinated subjects tested negative. Cross-verification with ELISA kits showed 34 positives in vaccinated samples by ELISA, aligning with the method’s reliability for both vaccinated and unvaccinated cohorts (Figure 8B) [121].

Sweat, a non-invasive biofluid, holds the potential for monitoring biomarkers linked to health and metabolism. For real-time monitoring in point-of-care testing (POCT), wearable devices offer an effective approach, particularly for detecting biomarkers in sweat. SERS-based sensors can identify molecules such as urea, uric acid, lactic acid, lactate, amino acids, and glucose with high specificity due to the unique Raman spectral fingerprints of each analyte [122,123,124,125]. However, the standalone SERS nanoprobe technique operates by detecting the SERS signals of individual nanoprobes, which poses challenges for wearable applications aimed at recognizing dynamically released target molecules in sweat. Instead, SERS active substrates are more suitable, as they can be integrated into wearable platforms and engineered to capture and enhance the Raman signals of biomarkers present in sweat.

Urine-based diagnostics offer a non-invasive, accessible way to detect a range of health indicators, including metabolites, toxins, and illicit drugs. The study by Koo et al. introduces a novel SERS nanotag-based assay for multiplexed detection of RNA biomarkers in prostate cancer (PCa) using urine samples (Figure 8C) [126]. This platform combines isothermal reverse transcription-recombinase polymerase amplification (RT-RPA) with SERS nanotags for rapid, sensitive, and specific detection, achieving a detection limit of 200 zmol. The assay identifies multiple biomarkers, including TMPRSS2 fusion genes, PCA3, and ARV7, which are critical for PCa subtyping and assessing treatment resistance. By using distinct Raman-active molecules attached to gold nanoparticles, each biomarker produces a unique spectral peak, allowing simultaneous detection without complex data processing. The technique has shown clinical applicability in both urine and tissue samples, offering a promising approach for non-invasive cancer diagnostics and personalized treatment strategies.

Exhaled breath analysis offers a non-invasive approach to disease detection, as it contains volatile organic compounds (VOCs) that can serve as biomarkers for conditions such as respiratory infections, diabetes, and cancer. In SERS-based breath diagnostics, substrates rather than standalone SERS nanoprobes are used due to the unique challenges of gas-phase sensing. Unlike liquid samples, where nanoprobes directly interact with analytes, detecting VOCs in exhaled breath requires stable platforms that effectively capture and retain gaseous molecules. Advanced SERS substrates, including metal–organic frameworks (MOFs) [127] and layered double hydroxides (LDH) [128], enhance VOC adsorption, facilitating stronger interaction with the SERS-active surface. This substrate-based approach is essential for detecting disease-specific VOCs and advancing non-invasive diagnostics for conditions like lung cancer.

#### 4.1.2. Cancer Detection

**Figure 9:**
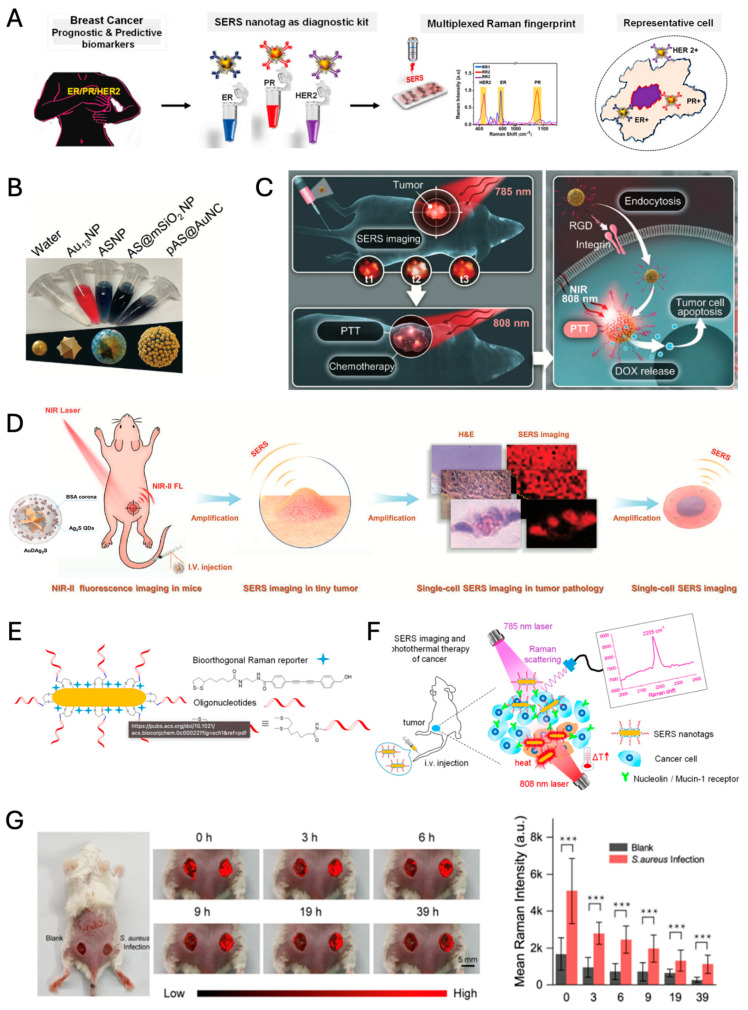
(**A**) Multiplexed biomarker detection using ER, PR, and HER2 IgGs conjugated SERS nanotags (adapted with permission from [130]; Copyright 2023 Elsevier). (**B**) Colors of Au_13_NPs, ASNPs, AS@mSiO_2_ NPs, and pAS@AuNCs suspended in nanopure water with SPR peaks at 518, 700, 734, and 806 nm, respectively (adapted with permission from [131]; Copyright 2023 Wiley-VCH). (**C**) Schematic illustration application of the multilayered mesoporous Au nanoarchitecture (RGD/DOX-pAS@AuNC) labeled with Raman reporter (MBA) via Au–thiol covalent bond for surface-enhanced Raman scattering (SERS) imaging-guided synergistic therapy toward cancer. (adapted with permission from [131]; Copyright 2023 Wiley-VCH). (**D**) Schematic illustration showing that AuDAg_2_S nanoprobes equipped with SERS/NIR-II optical imaging could multidimensional tumor images from living subjects, pathology to the single-cell and further guided NIR-II deeper photothermal therapy (adapted with permission from [132]; Copyright 2022 Wiley-VCH). (**E**) Fabrication of Oligonucleotide Modified Bioorthogonal SERS Nanotags (adapted with permission from [133]; Copyright 2020 American Chemical Society). (**F**) Bioorthogonal SERS nanotags as a precision theranostic platform for cancer detection and photothermal therapy in mice after intravenous injection (adapted with permission from [133]; Copyright 2020 American Chemical Society). (**G**) Photographic image of a BALB/c mouse with blank and *S. aureus* infected wounds after applying ACPA and SERS images at 2086 cm^−1^ of *S. aureus* (right) and blank (left) infected wounds at different time points (left). Corresponding average SERS intensities of ACPA on wounds. *** *p* < 0.001 (right) (adapted with permission from [134]; Copyright 2023 American Chemical Society).

SERS nanotags offer a versatile approach for cancer detection and theragnostic applications by targeting specific biomarkers. For instance, miR-29a has been identified as a potential biomarker for various cancers, including colorectal, breast cancer, hepatocellular carcinoma, acute myeloid leukemia, and lung cancer [129]. Additionally, relevant breast cancer biomarkers include estrogen receptor (ER), progesterone receptor (PR), and human epidermal growth factor receptor2 (HER2) [130]. The simple sandwich complex of the magnetic nanoparticle (MNP)/miR-29a/SERS nanotag (here, 4-mercapto benzoic acid modified Au nanorod) was demonstrated in determining the concentration of target miRNAs via magnetic separation, with the lowest detection of concentration 10 pM [129]. The multiplexing capability of the SERS nanotags (characteristic Raman reporters modified 40 nm Au NPs) has been demonstrated for the simultaneous recognition of ER, PR, and HER2 in breast cancer cell lines and tissue samples, offering a rapid and accurate method for prognostic analysis (Figure 9A) [130].

The multiplexing capacity of SERS tags endows them with the potential to act as multifunctional theragnostic platforms, enabling precision-targeted cancer diagnostics and the concurrent deployment of combined modality treatments, such as chemotherapy coupled with photothermal therapy. The platform comprises an Au NS core with a mesoporous silica and gold nanocluster (Au NC) shell, creating numerous hot spots that amplify SERS signals and enhance photothermal efficiency (Figure 9B,C). Loaded with the chemotherapeutic drug doxorubicin (DOX) and tagged with a Raman reporter (i.e., 4-mercaptobenzoic acid; MBA), the nanoprobe allows precise SERS imaging, enabling targeted drug delivery and real-time monitoring. This structure not only provides high SERS sensitivity for tracking but also achieves synergistic cancer cell destruction through localized heat and drug release, showing potential for non-invasive, targeted cancer treatments [131].

He et al. present an advanced SERS/NIR-II optical nanoprobe, Au NSs, DTTC (3,3′-Diethylthiatricarbocyanine iodide) Raman molecular tags, and silver sulfide quantum dots (NIR-II fluorophores) through a silica intermediate layer (named as AuDAg_2_S), designed for multidimensional tumor imaging and PTT, integrating SERS imaging and NIR-II fluorescence for deep-tissue visualization and precise tumor targeting (Figure 9D) [132]. Similar to the RGD/DOX-pAS@AuNC structure, AuDAg_2_S uses a layered design with an Au NS core and multiple coatings, enhancing its sensitivity and photothermal efficiency. However, while RGD/DOX-pAS@AuNC primarily focuses on SERS-guided chemo-PTT, the AuDAg_2_S nanoprobe combines NIR-II fluorescence with SERS, providing superior penetration for deep-tissue imaging up to 1 cm, significantly expanding its application in in vivo imaging. The high photothermal conversion efficiency (67.1%) and compatibility with NIR-II wavelengths enable precise, minimally invasive PTT for targeted cancer treatment. Both platforms highlight the multifunctionality of core–shell SERS-based nanoprobes, with AuDAg_2_S specifically extending imaging capabilities for complex biological structures and single-cell resolution.

Wang et al. demonstrate a bioorthogonal SERS nanotag with a Raman silent region reporter and aptamer-functionalized Au NR, allowing precise cancer cell targeting and effective photothermal ablation (Figure 9E,F) [133]. This unique bioorthogonal design allows for high-contrast SERS imaging in the Raman-silent spectral region, facilitating clearer differentiation of cancer cells within complex biological environments. By reducing background noise, this platform enhances detection specificity, allowing for both targeted imaging and effective photothermal ablation of cancer cells. This approach highlights a novel precision technique in SERS-based cancer theranostics, emphasizing molecular specificity and imaging clarity over the broader imaging depth or therapeutic synergies seen in the other studies.

#### 4.1.3. Monitoring of Chronic Diseases

Guo et al. detail the clinical relevance of aptamer-functionalized silver@cupriferous Prussian blue nanostructures (ACPA) in treating chronic wounds, utilizing their potential for SERS bioimaging (Figure 9G) [134]. ACPA’s innovative design permits a controlled release of silver, copper, and iron ions upon NIR light exposure, resulting in efficient anti-pathogenic action and promotion of cell migration and revascularization—all critical factors in chronic wound healing. Notably, the study underscores the method’s precision in detecting residual bacteria in wounds, with a remarkable LOD of 10 CFU (colony-forming unit)/mL for *Staphylococcus aureus* (*S. aureus*), enhancing the prospects for real-time, non-invasive clinical monitoring of infections. ACPA’s robust bactericidal activity, coupled with its ability to foster tissue regeneration, paves the way for advanced therapeutic interventions in diabetic wound care, while its high LOD signifies a significant advancement in diagnostic capabilities [134].

In summary, the integration of SERS technology into various diagnostic and therapeutic platforms offers a multifaceted approach to disease detection and treatment, with the potential for significant advancements in healthcare outcomes.

### 4.2. Food Applications

In the face of growing concerns regarding food safety and quality, along with their associated risks, food analysis has emerged as a significant global issue. This challenge has far-reaching implications, affecting the economic stability of the food supply chain and the health of consumers worldwide. Consequently, there is an intensified demand to innovate and develop precise methodologies for the detection of contaminants present in the food chain. SERS nanotags represent a significant advancement in this area, providing improved sensitivity and specificity for the detection of trace contaminants and potentially enhancing the standards of food safety testing.

**Figure 10:**
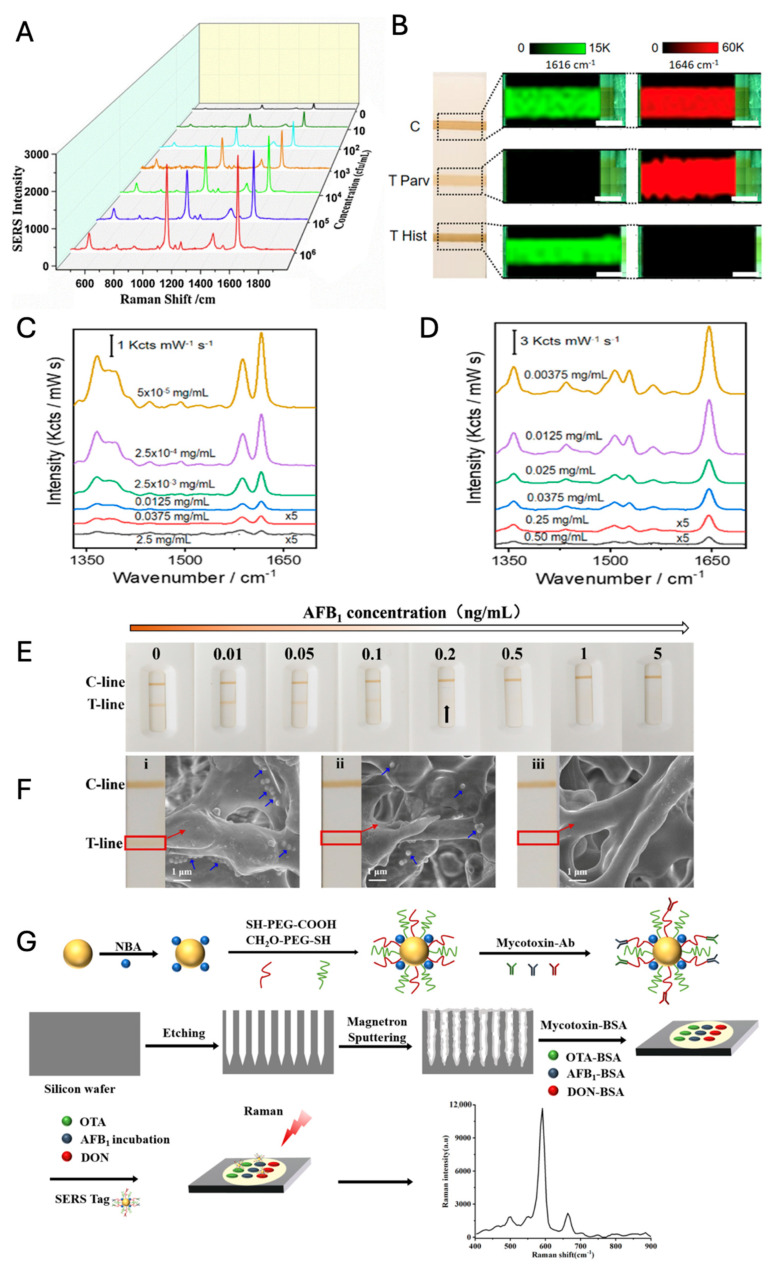
(**A**) SERS spectral responses obtained from the reaction of the developed SERS aptasensor with various concentrations of pathogens (adapted with permission from [135]; Copyright 2020 Elsevier). (**B**) Photographs of the LFIA strip with histamine (Hist), parvalbumin (Parv), and protein-G (PG) immobilized in the test (T) and control (**C**) lines, as indicated. SERS intensity mappings acquired at 1616 and 1646 cm^−1^, which are characteristic peaks of αHist-MGITC SERS or αParvRBITC SERS tags, respectively. (**C**) Average SERS spectra acquired from the different concentrations of histamine. (**D**) Average SERS spectra acquired from the different concentrations of Parvalbumin ((Figure 7B–D) adapted with permission from [136]; Copyright 2024 American Chemical Society). (**E**) Photographs of competitive LFIA (CLFIA) strips at different concentrations of AFB_1_. The black arrow marks the T-line, indicating the visible LOD (i.e., 0.2 ng/mL) as determined by 12 independent users using only the naked eye. (**F**) Photographs (left) and SEM images (right) of the CLFIA strip membrane at the AFB_1_ concentrations of (**i**) 0 ng/mL, (**ii**) 0.05 ng/mL, and (**iii**) 0.2 ng/mL. The blue arrows annotate Au-Ag alloy NPs-incorporated silica spheres captured in the T-line, with their number gradually decreasing as AFB_1_ concentration increases. No nanoparticles are observed in (**iii**). ((**E**,**F**) adapted with permission from [139]; Copyright 2023 American Chemical Society). (**G**) Scheme of the SERS microarray immunoassay for multiple mycotoxins (adapted with permission from [140]; Copyright 2024 American Chemical Society).

Duan et al. developed an aptamer–Eu complex ligated AuNP dimer for the sensitive and highly specific detection of *Shigella sonnei* (*S. sonnei*) in food samples, utilizing an aptamer-based target binding strategy [135]. This method showcased an impressive LOD of 10 CFU/mL for *S. sonnei*, far surpassing traditional polymerase chain reaction (PCR) and aptamer-based fluorescence assays (Figure 10A).

In the study by Pastoriza-Santos et al., a multiplex competitive LFIA leveraging Au@Ag core-shell NPs was employed for the simultaneous detection of histamine and parvalbumin in fish samples (Figure 10B–D) [136]. Two distinct types of Au@Ag SERS nanotags, each embedded with rhodamine B and malachite green, respectively, have been synthesized to enable the multiplexed detection of histamine and parvalbumin. These SERS nanotags achieved LODs (IC_10_) of 6.29 × 10^−5^ mg/mL for histamine and 7.74 × 10^−3^ mg/mL for parvalbumin, with quantification ranges established using IC_20_–IC_80_ criteria [137,138].

Wang et al. innovated with gold-silver alloy nanoparticles within a dendritic mesoporous silica scaffold to intensify the colorimetric label for sensitive competitive LFIA (Figure 10E,F) [139]. The amalgamation of alloy units with the dendritic structure resulted in a significant increase in the molar extinction coefficient and a collective signal effect, setting a new benchmark for the detection of aflatoxin B1 with an impressive LOD of 0.00314 ng/mL. This represents a significant improvement in sensitivity over traditional colorimetric LFIAs, highlighting the pivotal role of material engineering in enhancing SERS nanotag performance.

Li et al. adopted a different strategy by fabricating a highly active 3D SERS substrate with silver nanoparticles on porous silicon, which provided a large internal area and controllable pore size (Figure 10G) [140]. This substrate achieved high reproducibility and uniformity, which are crucial for reliable SERS-based assays. The target molecules were aflatoxin (AFB_1_), ochratoxin A (OTA), and deoxynivalenol (DON) as mycotoxins, which are toxic secondary metabolites produced by fungi and contaminated foods such as nuts, cereals, coffee beans, and dried fruits. Their microarray immunoassay for multiple mycotoxins displayed an extensive linear detection range and pg/mL levels of LOD, reinforcing the versatility of SERS nanotags in high-throughput analytic applications.

In conclusion, the application of SERS nanotags in food safety testing provides a multifaceted approach to detecting food contaminants with greater sensitivity and specificity. This advancement not only enhances food safety but also supports the economic stability of the food supply chain and protects public health. The continued development and refinement of these technologies hold great promise for the future of food safety testing, ensuring that consumers are better protected from the risks associated with foodborne contaminants.

### 4.3. Environmental Application

SERS nanotags also play a pivotal role in environmental monitoring, providing highly sensitive detection of pollutants at low concentrations. SERS sensors harness the power of metallic nanostructures to amplify Raman signals, enabling precise identification and quantification of environmental contaminants in water, soil, and atmosphere.

#### 4.3.1. Water

**Figure 11:**
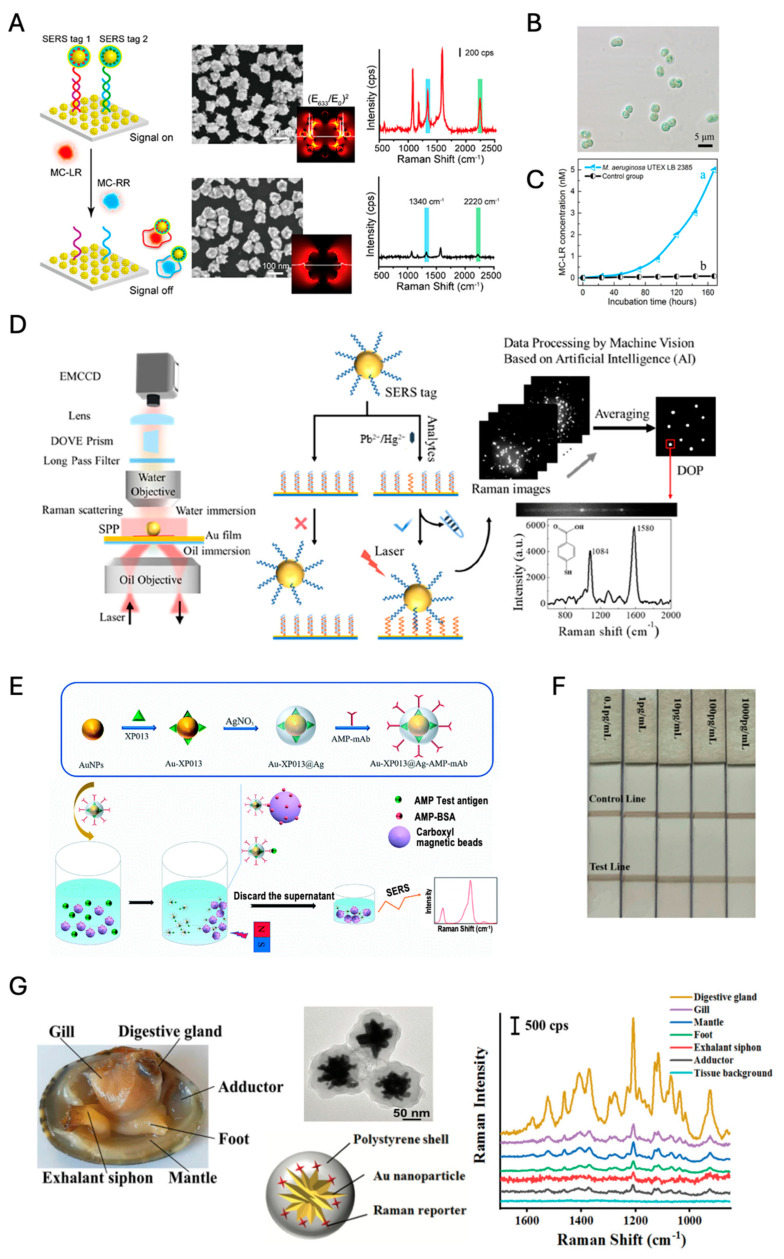
(**A**) Aptamer-based turn-off dual SERS sensor with AuNF-Au@tag@Ag@Au NP core-satellite assembly platform for MC-LR and MC-RR (L: leucine, R: arginine). (**B**) Optical brightfield image of *M. aeruginosa* UTEX LB 2385 cells. (**C**). MC-LR levels produced by *M. aeruginosa* UTEX LB 2385 (curve a) and *C. reinhardti* (curve b) over 7 consecutive days, as determined by the aptasensor ((**A**–**C**) adapted with permission from [141]; Copyright 2021 American Chemical Society). (**D**) Schematic diagram of the optical setup of the SPR-SERS microscope and detecting strategy for Pb^2+^ and Hg^2+^ using single-particle Raman imaging (adapted with permission from [142]; Copyright 2023 American Chemical Society). (**E**) SERS-based AMP immunoassay with magnetic separation (adapted with permission from [143]; Copyright 2022 Royal Society of Chemistry). (**F**) Detection of series BPA actual samples using the SERS ICA (ICA: immunochromatographic assay) strips (adapted with permission from [144]; Copyright 2022 Elsevier). (**G**) An image of the detected organs of a bivalve *Ruditapes philippinarum*, Au NS@polystyrene (PS) core@shell structures with Cy7 dyes, and typical SERS spectra measured from the organs of the clams exposed to SERS@PS for 24 h. (adapted with permission from [145]; Copyright 2022 Royal Society of Chemistry).

In water bodies, it is crucial to assess the presence of specific pollutants that can impact both ecosystem and human health. Detecting microcystins (MCs), a common toxin often found in these environments, is especially important. Masson et al. developed SERS nanotags using a core–satellite structure comprising Au NPs at the core, encapsulated by an Ag shell [141]. This configuration was then functionalized with aptamers targeted at detecting microcystins (MCs), a class of toxins commonly found in water bodies. The design of these nanotags allows for an effective EM field enhancement around the nanoparticles, leading to a lower LOD of just 0.1 ng/mL for MCs in aquatic environments (Figure 11A–C). The aptamer-functionalized nanotags offer a robust sensing strategy where the presence of MCs alters the SERS signal, enabling quantitative analysis that is crucial for assessing water safety.

Simultaneously, it is essential to detect heavy metals such as lead (Pb^2+^) and mercury (Hg^2+^) in water systems, as these toxic elements pose significant risks to both environmental and human health. Zhu et al. approached the design of SERS nanotags by utilizing Au NPs modified with ion-responsive functional nucleic acids (i.e., aptamers or nanozymes) [142]. A custom-built coaxial dual-objective microscope system was employed to achieve single-particle analysis with wide-field Raman imaging (Figure 11D). Analyzing the number density of SERS-tagged particles with the aid of artificial intelligence (AI) can be more advantageous for the quantitative analysis of analytes than measuring the overall SERS signal of the particles because it is less affected by imbalances in the signal distribution of SERS-tagged particles. Their technique enables the selective detection of heavy metals like Pb^2+^ and Hg^2+^, with an impressively low LOD of 1 pM. Remarkably, the technique has demonstrated robust performance in real-world sample analyses (i.e., determination of Hg^2+^ and Pb^2+^ spiked in lake and river water), offering high recovery rates and the capability to perform multianalyte detection within complex environmental matrices.

In a notable study by Guan et al., a novel approach was developed for the detection of amphetamine (AMP) using SERS-based competitive immunoassay coupled with magnetic separation [143]. The method utilized Au-Ag bimetallic nanoparticles (Au-XP013@Ag) as SERS substrates to enhance Raman signals. The use of magnetic beads for antigen labeling and antibodies attached to SERS nanotags facilitated a competitive binding mechanism, significantly improving the detection specificity and reducing the LOD to 2.28 ng/mL (Figure 11E). The setup demonstrates a portable application potential, especially in point-of-care environmental testing.

Liang et al. presented a rapid and highly sensitive method for detecting trace amounts of bisphenol A (BPA) in water using a combination of immunochromatographic detection technology and SERS [144]. Their method utilizes a competitive immunoassay format where an anti-BPA antibody is conjugated to Au(core)@Ag(shell) nanoparticles, serving as a SERS nanoprobe in a rapid LFIA platform. Notably, the detection limit achieved is exceptionally low at 0.1 pg/mL, which significantly surpasses the capabilities of traditional methods like gas chromatography and liquid chromatography (Figure 11F).

Chen et al. quantitatively investigated nanoplastic distribution in the marine bivalve *Ruditapes philippinarum* using SERS nanotags and inductively coupled plasma mass spectroscopy (ICP-MS) (Figure 11G) [145]. The research reveals that after a three-day exposure period, a substantial 86.7% of nanoplastics accumulate predominantly in the digestive gland of the bivalve, significantly more than in other organs like the gills and mantle. Impressively, the digestive gland was capable of excreting 98.7% of these nanoplastics following an 11-day depuration period, although other organs showed much lower clearance rates. Their study highlights the persistence and potential trophic transfer of nanoplastics within marine food webs, indicating substantial human health risks from the consumption of nanoplastic-contaminated seafood.

#### 4.3.2. Soil

In environmental monitoring, accurately detecting and quantifying soil pollutants, such as pesticides, heavy metals, and growth regulators, is crucial for safeguarding food security, ecosystem health, and human well-being. Traditional sensors, including electrochemical and fluorescence-based sensors, are widely used for soil analysis but often face limitations in sensitivity, specificity, and interference from complex soil matrices. SERS sensors offer a promising alternative, leveraging plasmonic nanostructures to amplify weak Raman signals of target molecules, thereby achieving high sensitivity and specificity even at trace levels. SERS sensors also enable rapid, on-site detection without extensive sample preparation, making them ideal for monitoring persistent pollutants like paclobutrazol and heavy metals in soil.

Paclobutrazol, a widely used growth regulator to enhance crop yields, has a stable presence in soil, which raises environmental and health concerns due to its potential carcinogenicity and detrimental effects on soil microbiota. As such, detecting paclobutrazol residues in soil is critical. To this end, FSAA-MIP SERS sensors—comprising a molecularly imprinted polymer (MIP)-Fe_3_O_4_@SiO_2_-Au@Ag (FSAA) structure—have been developed [146]. In this approach, soil samples are mixed with FSAA-MIP and incubated for two hours, after which the sensors are separated and analyzed using Raman spectroscopy. These sensors demonstrate high specificity and selectivity, achieving a detection limit of 0.075 μg/g, and exhibit minimal interference from other soil contaminants and organic pollutants, thanks to their “key-and-lock” recognition mechanism.

**Figure 12:**
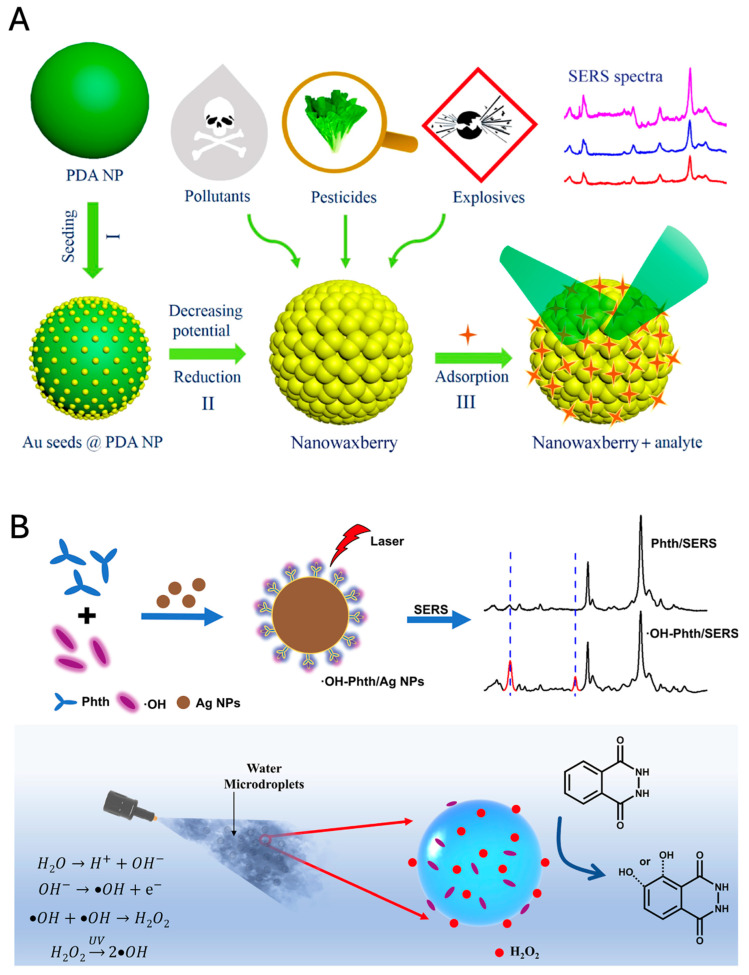
(**A**) Schematic illustration of the synthesis process of polydopamine@gold (PDA@Au) nanowaxberry and its SERS detection. (I) Deposition of Au seeds onto the surface of the PDA sphere, (II) the iodide ions assisted the growth of Au nanoshell on the PDA sphere, and (III) SERS detection of pesticides, pollutants, and explosives using nanowaxberry as a substrate (adapted with permission from [147]; Copyright 2018 American Chemical Society). (**B**) Schematic of the SERS nanosensor for •OH detection and mechanism and detection of H_2_O_2_ and •OH generation in water microdroplets (adapted with permission from [148]; Copyright 2024 American Chemical Society).

The polydopamine@gold (PDA@AuSERS) sensor, known as “nanowaxberry,” was developed to detect various pollutants, including thiram, benzidine, and 2,4-dinitrotoluene (DNT), in complex samples, as shown in Figure 12A [147]. In this approach, a colloidal nanowaxberry solution mixed with analytes is applied onto silicon slices, air-dried, and analyzed for Raman signals. This sensor achieved a detection limit of 0.31 μg/g for thiram in soil, well below the maximum residue limits (0.1–5.0 mg/kg) set by China and the European Union. Despite some interference from soil organic carbon, the characteristic Raman peaks of thiram remained clear. Additionally, it effectively detected benzidine in environmental water with a detection limit of 100 nM (0.018 ppm), surpassing the maximum allowable levels in drinking water (0.200 ppm). The nanowaxberry sensor also showed sensitivity for DNT detection in water. This sensor combines high SERS sensitivity with excellent reproducibility, making it a promising tool for environmental monitoring.

#### 4.3.3. Atmosphere

Atmospheric microdroplets, such as clouds, fog, and aerosols, are key environments where many aqueous chemical reactions take place, impacting pollutant transformation and atmospheric cleansing processes. Studying these microdroplets can enhance our understanding of pollutant degradation, particularly through the formation of reactive species like hydroxyl radicals (•OH) and hydrogen peroxide (H_2_O_2_). In this context, a novel SERS nanosensing system, composed of phthalhydrazide (Phth) with Ag NPs, has been introduced as a highly selective and sensitive probe specifically for detecting •OH radicals in atmospheric water microdroplets (Figure 12B) [148]. The Phth probe reacts quickly with •OH, resulting in measurable SERS spectral changes that indicate structural shifts. This system achieves a detection limit of 0.34 nM for •OH and demonstrates strong selectivity, with minimal interference from other reactive oxygen species (ROS) such as H_2_O_2_. As such, it offers a valuable tool for advanced studies on the formation of H_2_O_2_ in the atmosphere, contributing to our understanding of environmental oxidation processes and their broader implications for air quality and ecological health.

Finally, the application of SERS nanotags in environmental monitoring offers a powerful tool for detecting pollutants with high sensitivity and specificity. These advancements not only facilitate early detection and intervention but also contribute to the broader goal of safeguarding environmental and public health. The continued development and deployment of SERS-based technologies promise to elevate standards in environmental monitoring and provide critical insights into pollutant dynamics and their impacts.

### 4.4. Challenges of SERS Nanoprobes for Real Applications

SERS nanoprobes hold immense potential for highly sensitive molecular detection in applications such as biomedical diagnostics, environmental monitoring, and food safety. With their ability to amplify molecular “fingerprints” and provide highly specific data, SERS nanoprobes have emerged as powerful analytical tools. However, realizing the full practical utility of SERS nanoprobes involves overcoming significant challenges related to stability, reproducibility, and data interpretation. Each of these challenges plays a critical role in advancing SERS technology from experimental research to reliable, real-world applications.

#### 4.4.1. Structural and Signal Stability

Stability is fundamental for the consistent performance of SERS nanoprobes, with two primary dimensions: structural stability and signal stability. Structural stability concerns the physical and chemical robustness of the nanoprobes, which must withstand a variety of environmental factors without degradation. In biomedical applications, SERS nanoprobes encounter challenging intracellular environments, including acidic conditions within lysosomes (as low as pH = 4.5~5.5), high ionic strengths, elevated enzymatic activity, and the presence of reactive oxygen species (ROS). For instance, the low pH within lysosomes promotes protonation and can accelerate the degradation of certain nanomaterials, particularly silver-based SERS nanoprobes. Additionally, the low pH can also promote ligand desorption from metal surfaces and shift the charge of normally negatively charged ligand molecules toward a more positive state, reducing electrostatic repulsion and thereby compromising nanoprobe stability. High ionic strengths in physiological fluids can weaken electrostatic repulsion, promoting nanoprobe aggregation and reducing the stability of the nanoscale “hot spots” necessary for enhanced Raman signals. ROS can oxidize silver-based nanoprobes, compromising their structural integrity and reducing their effectiveness over time.

In environmental applications, stability challenges arise from varied exposure conditions in soil, water, and air. Soil matrices, for example, contain organic compounds, diverse pH levels, and microbial activity that can lead to nanoprobe aggregation or chemical degradation. Aquatic environments add complexity through factors like salinity, which neutralizes surface charges and promotes nanoparticle aggregation, limiting available surface area for analyte interaction. In airborne settings, SERS nanoprobes are exposed to fluctuating temperatures, UV radiation, and oxidative pollutants, all of which can degrade structural integrity.

To mitigate these issues, protective coatings such as polyethylene glycol (PEG) and silica have been applied to create a physical barrier, reducing oxidation and stabilizing nanoprobe dispersion. Surface engineering techniques such as polyvalent DNA modification, protein corona formation, and lipid immobilization have been widely used for the biomedical application of nanomaterials and nanostructures. Au–thiol bonds are commonly used in surface modifications of SERS nanoprobes because they form stable, covalent interactions between gold surfaces and thiol-containing ligands, including biomolecules. However, cellular environments present certain challenges that can affect the longevity and robustness of these bonds. ROS, such as superoxide and hydrogen peroxide, can oxidize thiols, leading to potential bond cleavage or detachment from the gold surface, as described before. In the cytosol, primarily due to high concentrations of glutathione, a tripeptide with a thiol group can bind to gold surfaces. Glutathione can displace weaker-bound thiol ligands through a ligand exchange reaction, potentially disrupting the original functionalization of the nanoprobe. High intracellular levels of glutathione can, therefore, compete with functional thiol ligands on the nanoprobe surface, leading to ligand desorption and reduced stability of the gold-thiol bond. To enhance the resilience of the Au–S bond in cellular environments, several strategies can be employed, such as the introduction of hydrophobic spacers between the thiol and the PEG or terminal group [149,150], protective polymer coatings [151,152], and multidentate thiol-based anchoring [153,154].

Signal stability focuses on achieving a consistent SERS response across measurements. Variability in signal intensity, caused by factors like fluctuating hot spot distribution and nanoprobe interactions with the surrounding medium, poses challenges for quantitative applications. In biological samples, dynamic conditions, such as shifts in pH or interactions with proteins, can disrupt hot spot formation, leading to inconsistent signals. In environmental samples, similar issues arise due to temperature fluctuations, organic matter deposition, and pH variability. These factors can impede reliable analyte quantification, which is essential for diagnostic or monitoring applications where precise and stable measurements are critical.

#### 4.4.2. Reproducibility and Reliability for Quantification

For SERS nanoprobes to be used as quantitative tools, achieving reproducible and reliable measurements is essential. Variations in signal intensity, substrate quality, and hot spot distribution complicate the quantification of analytes, especially across different laboratories and instruments. Consistent and accurate measurements are particularly challenging in SERS because minor changes in nanoparticle arrangement or experimental setup can cause significant variations in signal output [155,156].

To improve reproducibility, recent studies have emphasized the importance of using internal standards, such as well-characterized gold nanoparticle tags, to normalize signals across measurements. These internal standards account for variations in experimental conditions and help standardize SERS intensity, improving reliability for quantitative analysis. Additionally, incorporating calibration models that account for variables like nanoparticle concentration and hot spot distribution can enhance signal quantification and enable more robust data interpretation. Multivariate statistical methods, such as principal component analysis and Gaussian binning, have also proven effective in managing signal variability and reducing noise, thereby enabling SERS to be used more consistently in complex sample matrices [157,158].

Interlaboratory studies have shown that harmonizing standard operating procedures (SOPs) and implementing uniform calibration standards are crucial for achieving reproducibility across different research groups. By adhering to standardized protocols and thoroughly characterizing SERS substrates, researchers can reduce experimental variability and improve the reliability of results. This standardization is particularly important for applications that require absolute accuracy, such as clinical diagnostics and environmental monitoring, where quantification precision is essential [156,158].

#### 4.4.3. Artificial Intelligence for Enhanced Data Interpretation

Data interpretation is a critical aspect of SERS, as each spectrum carries complex, high-dimensional information that reflects the molecular composition of the sample. In practical applications, especially those involving large datasets or high-throughput analysis, manually interpreting SERS data can be both time-consuming and error-prone. Artificial intelligence (AI) and machine learning (ML) have shown great promise in automating data analysis, improving accuracy, and enhancing reproducibility in SERS applications [159].

Machine learning algorithms are particularly effective at pattern recognition, peak identification, and noise reduction in SERS spectra, facilitating the rapid and accurate detection of analytes. By analyzing historical datasets, AI models can learn from past trends and adjust their calibration, making them adaptive and resilient in diverse experimental conditions. For example, ML algorithms have been employed to distinguish between molecular signatures in complex biological samples, which aids in biomarker identification and real-time diagnostics. This integration of AI not only automates spectral processing but also helps compensate for variabilities in experimental conditions, thereby enhancing data reliability and consistency across different instruments and setups [156,160].

AI-augmented SERS platforms are particularly useful in applications that require quick decision-making, such as point-of-care diagnostics, environmental pollutant screening, and food contamination monitoring. These AI-driven systems can process large volumes of spectral data in real time, facilitating rapid detection and classification of contaminants or biomarkers. By incorporating AI, SERS technology can achieve the high-throughput capability necessary for field-deployable and clinical applications, transforming it into a robust and adaptable analytical tool for complex environments [159].

## 5. Conclusions

Standalone SERS nanoprobes mark a significant advancement in SERS applications such as analytical chemistry, biomedicine, and environmental science. Sophisticated designs such as nanogap and core–satellite structures in standalone SERS nanoprobes have augmented the essential sensitivity and specificity for single-molecule detection in complex matrices. In biomedical diagnostics, SERS nanoprobes facilitate the early detection of disease biomarkers, operating effectively in complex biological conditions. Environmental monitoring also benefits from these nanoprobes, which detect low levels of toxins and pollutants, aiding in timely interventions to prevent environmental damage. Furthermore, in food safety, their ability to detect multiple contaminants simultaneously improves the efficiency of quality control processes. The performance and characteristics of SERS nanotags for various applications are summarized in Table 2.

However, challenges such as probe stability, reproducibility, and the need for standardized protocols remain unsolved. The standalone SERS nanotag system offers significant quantitative advantages over direct SERS methods and target-mediated SERS signal variation methods due to its robust and stable SERS signal. Ensuring superior particle stability in biologically and environmentally harsh conditions is essential for reliable, quantitative SERS analysis. Employing multidentate ligands [154,168,169,170,171] and innovative ligands, such as cyclic PEG molecules [169,172,173], along with advanced ligand engineering techniques [150,174,175,176,177], can significantly enhance the binding affinity and functionality of nanoparticles, outperforming common ligands in maintaining stability and performance [178,179,180,181]. To address the inherent reproducibility issues of SERS techniques, ratiometric SERS techniques [182,183,184,185,186,187] using internal standard SERS molecules and emerging digital Raman analysis [188,189,190] are proving effective for practical applications with accurate and reliable quantification.

Addressing these challenges is crucial for the broader adoption of SERS nanoprobes, particularly in developing cost-effective, user-friendly portable devices for low-resource settings. Future research should focus on refining the technological aspects of SERS nanoprobes and developing standardized methods for their production and application. This will enable their integration into practical tools for diagnostics and environmental monitoring, thereby expanding their role in advancing scientific research and public health.

## Figures and Tables

**Table 1:** Advantages and disadvantages of various nanostructures for SERS nanotags.

NP Types		Advantages	Disadvantages	Optimization Strategies	Refs.
Simple solid	Isotropic (sphere, cube, etc.)	- Simplified preparation - Relatively high structural uniformity - Cost-effective	- Low SERS performance	- Design of sharp, high-edged structures - Prevention of SERS dye desorption	[69,71,72]
	Anisotropic (rod, star, etc.)	- High tunability - Relatively high SERS intensity	- Relatively complicated preparation - Relatively low signal uniformity	- Elaborate tuning control (e.g., aspect ratio) - Control of SERS dye positioning - Prevention of SERS dye desorption	[63,67]
Inter-nanogap		- Highly enhanced EM field within nanogap - Excellent SERS performance	- Sophisticated preparation - Low synthetic yield - Concomitant multimeric structures - Light orientation angle dependence - Expensive	- Elaborate synthesis of uniform nanogap structures - Ensuring nanogap uniformity - Structural robustness of nanogap during reaction - Accurate positioning of SERS dyes within nanogaps - Discrimination of other multimeric structures	[79,81,89,90]
Core–shell		- High tunability in design and function - Moderate structural uniformity - Relatively easy synthesis compared to nanogapped structures	- Lower SERS signal compare to nanogapped structures - Susceptible to shell degradation under etching conditions and extreme pH	- Lattice matching between core and shell to reduce strain and defects - Dense SERS dye embedding between core and shell - Shell thickness optimization	[51,52]
Core–satellite		- High tunability (i.e., size, density, material of satellites) - High SERS intensity	- Low signal uniformity - Complexed EM enhancement mechanism among core and satellites	- Uniformity of size, shape, and distribution of satellites - Control of naogap among core and satellites	[95,99,102]
Intra-nanogap		- Highly robust SERS signal - Highly enhanced EM field within nanogap - Excellent SERS performance - Facile and flexible surface modification	- Sophisticated preparation - Low synthetic yield - Expensive	- Elaborate synthesis of uniform nanogap structures - Ensuring nanogap uniformity - Embedding SERS dyes within intra-nanogaps	[106,110,114,115]

**Table 2:** A summary of SERS substrates for clinical, food, and environmental applications mentioned in this review.

**CLINICAL**				
**SERS Substrate**	**Target**	**Linear Range**	**LOD**	**Ref.**
Au NRs	TBI biomarkers	1 pg/mL to 50 ng/mL	∼10^−1^ pg/mL	[119]
SiO_2_@Au NRbs	IAV	2.63 × 10^3^ to 10^9^ copies/mL	2.63 × 10^3^ copies/mL	[120]
Ag^MBA^@Au NPs	SARS-CoV-2 IgG	10^−9^ to 10^−4^ mg/mL	0.22 pg/mL	[121]
RT-RPA/SERS	Prostate cancer tumor RNA biomarker	log(1–10^6^) copies	200 zmol (100 copies)	[126]
GSPs@ZIF-8	VOCs	-	10 ppb	[127]
Ag@LDH	VOCs (Aldehyde)	0 to 100 ppm	1.9 × 10^−9^ *v*/*v* (1.9 ppb)	[128]
Au NRs–4-MBA	miR-29a	0 to 1000 pM	10 pM	[129]
AuNP@CV@PEG@anti-ER/PR/HER2	ER/PR/HER2	(1^+^, 2^+^ and 4^+^ tissue SERS imaging)	Recognized of 1^+^, 2^+^ and 4^+^ tissue SERS imaging	[130]
RGD/DOX–pAS@AuNC	HeLa cells	(0 to 24 h SERS imaging)	Maximum 2 h SERS imaging	[131]
AuDAg_2_S	CT26 colon cancer cells	(0.25 to 24 h SERS imaging)	Maximum at 4 h SERS imaging	[132]
Au NR	MCF-7 cells	-	Recognized MCF-7 cells at 6 h.	[133]
Au@4-MBA@Ag	cTn I	0.01 to 10.0 ng/mL	0.0086 ng/mL	[161]
AuNPs	β-lactamases	10^3^ to 10^7^ cfu/mL	10^3^ cfu/mL	[162]
ACPA	*S. aureus*	10 to 10^9^ cfu/mL	10 CFU/mL	[134]
Fe_3_O_4_@DTNB@Au	Hyaluronidase	10^−3^ to 10 U/mL	0.32 mU/mL	[98]
Ag@MBN@ PEG-NH**_2_**	MMP-2	5 to 100 ng/mL	2.067 ng/mL	[103]
SiO_2_@Au-Ag Janus CJS	Carbohydrate antigen 19-9	3 × 10^−5^ to 1 × 10^4^ IU/mL	3.7 × 10^−5^ IU/mL	[97]
CS@SiO_2_	SARS-CoV-2	0 to 1000 PFU/mL	8.81 PFU/mL	[100]
AHETE dimer	microRNA	0.1 to 100 pM	0.011 amol/ngRNA	[85]
AHSBS dimer	microRNA		0.023 amol/ngRNA	
**FOOD**				
**SERS Substrate**	**Target**	**Linear Range**	**LOD**	**Ref**
citrate-stabilized Au nanoparticles (cit-Au NPs)	*S. sonnei*	10 to 10^6^ cfu/mL	10 cfu/mL	[135]
Au@Ag NPs	Histamine	2.5 to 5 × 10^−6^ mg/mL	6.29 × 10^−5^ mg/mL	[136]
	Parvalbumin	0.5 to 2.5 × 10^−4^ mg/mL	7.74 × 10^−3^ mg/mL	
Ti_3_C2Tx MXenes	AFB_1_	0.001 to 100 ng/mL	0.6 pg/mL	[163]
Au-Ag NPs	AFB_1_	0.01 to 0.2 ng/mL	0.00314 ng/mL	[139]
AgNP-Psi	OTA	0.001 to 10,000 ng/mL	3.35 pg/mL	[140]
	AFB_1_		0.36 pg/mL	
	DON		2.70 pg/mL	
Au@Ag NP	Patulin	0.05–250 ng/mL	0.0281 ng/mL	[99]
AuMBA@AgMBA-antigen and AuMBA@AgMBANPs	Zearalenone	5 to 400 μg/kg	3 μg/kg	[102]
MDAu@Ag-DTNB and MDAu@Ag-MBA	Kanamycin	0.15 pg/mL to 3 ng/mL	0.52 pg/mL	[164]
	Ractopamine (RAC)		2.5 pg/mL	
	Clenbuterol		6.2 pg/mL	
	Chloramphenicol		0.87 pg/mL	
Au NPs	Pb^2+^	10^−16^ to 2 × 10^−12^ M	-	[165]
Ag@4-MBN@Ag-c-DNA	Histamine	10^−2^ to 10^5^ ng/mL	0.65 × 10^−3^ ng/mL	[166]
Fe_3_O_4_@SiO_2_–Au-Apt	*Escherichia coli*	10^1^ to 10^8^ cfu/mL	10 cfu/mL	[167]
cGNPs-4MBA-cDNA	RAC	0.05 ng/mL to 10 μg/mL	0.03 ng/mL	[96]
**ENVIRONMENTAL**				
**SERS Substrate**	**Target**	**Linear Range**	**LOD**	**Ref**
Au@label@Ag@Au NPs	MC-LR	0.01 to 10 nM	1.5 pM	[141]
	MC-RR	0.01 to 10 nM	1.3 pM	
Au@Ag	BPA	0.1 to 1000 pg/mL	0.1 pg/mL	[144]
Au-XP013@Ag-AMP-mAb	AMP	0 to 200 ng/mL	2.28 ng/mL	[143]
Au NPs on Au film	Pb^2+^	100 pM to 100 nM	1 pM	[142]
	Hg^2+^		100 fM	
SERS@PS@BSA	Nanoplastics (in bivalve *Ruditapes* *philippinarum*)	0.2 mg/L (about 5.9 × 10^11^ particles per mL	-	[145]
Fe_3_O_4_@SiO_2_-Au@Ag (FSAA)	Paclobutrazol	0.075 to 12.75 μg/g	0.075 μg/g	[146]
PDA@AuSERS	Thiram	11 μg/g to 0.31 μg/g	0.31 μg/g	[147]
	Benzidine	10 μM to 100 nM	100 nM (0.018 ppm)	
	2,4-Dinitrotoluene DNT	-	Presence	
•OH-Phthalhydrazide (Phth)/AgNPs	•OH	2 nM to 2 μM	0.34 nM	[148]

## Data Availability

Data sharing is not applicable.

## Abbreviations

1:4-BDT	1,4-Benzenedithiol
4-MBN	4-Mercaptobenzonitrile
4-NBT	4-Nitrobenzenethiol
4-ATP	1,4-Aminothiophenol
ACPA	Prussian blue nanostructures
AFB_1_	Aflatoxin
Ag	Silver
AI	Artificial intelligence
Al	Aluminum
AMP	Amphetamine
Au	Gold
AuDGNs	Au dual-gap nanodumbbells
Au NC	Au nanocluster
Au-NNP	Au-nanogapped nanoparticle
AuNRs	Gold nanorods
Au NSs	Au nanostars
BPA	Bisphenol A
CA19-9	Carbohydrate antigen 19-9
CB	Conduction band
CFU	Colony-forming unit
CJS	Core-Janus satellite
CLE	Clenbuterol
CM	Chemical mechanism
CT	Charge transfer
Cu	Copper
DDA	Discrete dipole approximation
DNT	2,4-dinitrotoluene
DON	Deoxynivalenol
DOX	Doxorubicin
DTNBs	5,5′-dithiobis(2-nitrobenzoic acids)
*E. coli*	*Escherichia coli*
EF	Enhancement factor
ELISA	Enzyme-linked immunosorbent assay
EM	Enhancement Electromagnetic fields
ER	Estrogen receptor
ERA	Enzymatic recombinase amplification
ESBLs	β-lactamases
FDTD	Finite-difference time-domain
FEM	Finite-element method
Ga	Gallium
GCS	Glasgow Coma Scale
GERTs	Gap-enhanced Raman tags
GFAP	Glial fibrillary acidic protein
HAase	Hyaluronidase
HER2	Human epidermal growth factor receptor2
Hg	Mercury
HOMO	Highest occupied molecular orbital
IAV	Influenza A virus
ICA	Immunochromatographic assay
IU	International unit
ICP-MS	Inductively coupled plasma mass spectroscopy
Kana	Kanamycin
LDH	Layered double hydroxides
LF	Lateral flow
LFAs	Lateral flow assays
LFIA	Lateral flow immunoassay
LSPRs	Localized surface plasmon resonances
LOD	Low limit of detection
LUMO	Lowest unoccupied molecular orbital
MB	Magnetic bead
MBN	Mercaptobenzonitrile
MBP	Myelin basic protein
MCs	Microcystins
Mg	Magnesium
MIP	Molecularly imprinted polymer
ML	Machine learning
MMP-2	Metalloproteinase 2
MNP	Magnetic nanoparticle
MOFs	Metal–organic frameworks
NIR	Near-infrared
NPs	Au nanoparticles
NSM	Nanosnowman
O-GERTs	Orthogonal-GERTs
•OH	Hydroxyl radicals
H_2_O_2_	Hydrogen peroxide
OTA	Ochratoxin A
OXNCs	Open cross-gap (X-gap) nanocubes
PAT	Patulin
Pb	Lead
PCa	Prostate cancer
PCR	Polymerase chain reaction
PDA@AuSERS	Polydopamine@gold
PEG	Polyethylene glycol
PEI	Polyethyleneimine
P-GERTs	Petal-like external nanogaps
Phth	Phthalhydrazide
PLFSs	Paper lateral flow strips
POC	Point-of-care
POCT	Point-of-care testing
PR	Progesterone receptor
PS	Polystyrene
PTT	Photothermal therapy
RAC	Ractopamine
ROS	Reactive oxygen species
RT-RPA	Reverse transcription-recombinase polymerase amplification
SARS-CoV-2 IgG	Coronavirus-2 immunoglobulin G
*S. aureus*	*Staphylococcus aureus*
SEM	Scanning electron microscopy
SERS	Surface-enhanced Raman scattering
S-GERTs	Smooth external shell structures
SHINERS	Shell-isolated nanoparticle-enhanced Raman spectroscopy
SLN	Sentinel lymph node
SOPs	Standard operating procedures
*S. sonnei*	*Shigella sonnei*
TBI	Traumatic brain injury
TEM	Transmission electron microscopy
TiN	Titanium nitride
VB	Valence band
VOCs	Volatile organic compounds
ZEN	Zearalenone
